# When Identification of the Reduction Sites in Mixed Molybdenum/Tungsten Keggin-Type Polyoxometalate Hybrids Turns Out Tricky

**DOI:** 10.1021/acs.inorgchem.2c00866

**Published:** 2022-05-12

**Authors:** Maxime Laurans, Michele Mattera, Raphaël Salles, Ludivine K’Bidi, Pierre Gouzerh, Séverine Renaudineau, Florence Volatron, Geoffroy Guillemot, Sébastien Blanchard, Guillaume Izzet, Albert Solé-Daura, Josep M. Poblet, Anna Proust

**Affiliations:** ‡ Institut Parisien de Chimie Moléculaire, Sorbonne Université, CNRS, 4 Place Jussieu, F-75005 Paris, France; § Department de Química Física i Inorgànica, Universitat Rovira i Virgili, Marcel-lí Domingo 1, 43007 Tarragona, Spain

## Abstract

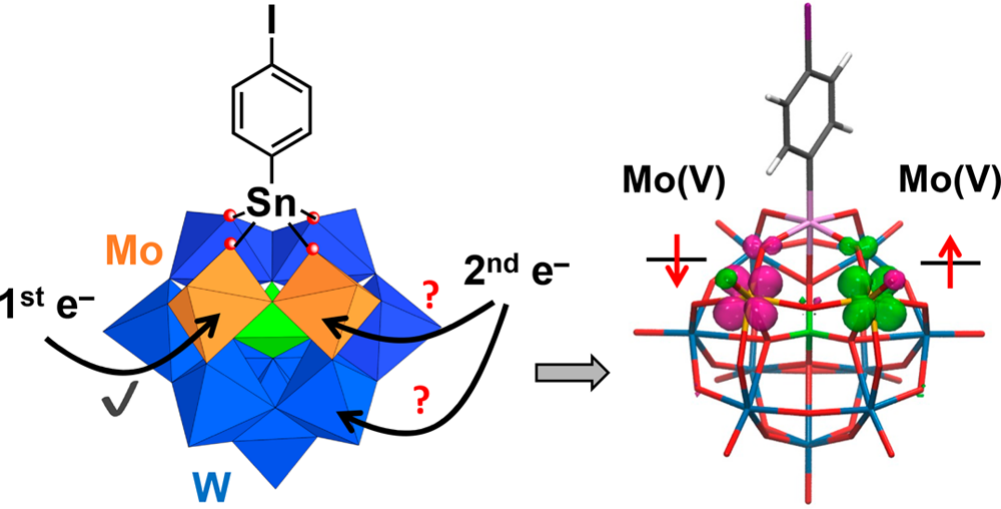

The mixed molybdenum/tungsten Keggin-type polyoxometalate (POM) hybrid (TBA)_4_[PW_9_Mo_2_O_39_{Sn(C_6_H_4_I)}] (TBA = *tert*-butylammonium) has been prepared by the reaction between [α-PW_9_Mo_2_O_39_]^7–^ and [Cl_3_Sn(C_6_H_4_I)] in dried acetonitrile, in the presence of tetra-*n*-butylammonium bromide. A further coupling reaction affords the ferrocenyl derivative (TBA)_4_[PW_9_Mo_2_O_39_{Sn(C_6_H_4_)C≡C(C_6_H_4_)Fc}]. The POM hybrids have been thoroughly characterized by NMR and IR spectroscopies. Electrochemical analysis confirms their ease of reduction compared to the all-W analogue, albeit with a second reduction process occurring at a lower potential than in the all-Mo species. It is noteworthy that the second reduction is accompanied by an unusual red shift of the electronic absorption spectrum. Whereas there is no doubt that the first reduction deals with Mo, the location of the second electron in the bireduced species, on the second Mo or on W, has thus been the subject of a cross-investigation by spectroelectrochemistry, electron spin resonance, and theoretical calculations. Finally, it came out that the second reduction is also Mo-centered with two unpaired and antiferromagnetically coupled extra electrons.

## Abstract 2

The sites for the successive reduction processes of a mixed molybdenum/tungsten Keggin-type organotin hybrid are debated through a combination of spectroelectrochemical investigations and theoretical calculations.

## Introduction

**Scheme 1:**
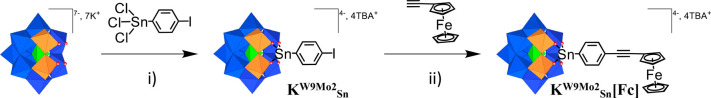
Synthetic Routes to the Mixed Mo/W POM Hybrids **K**^**W9Mo2**^_**Sn**_ and **K**^**W9Mo2**^_**Sn**_**[Fc]** In this representation, WO_6_, MoO_6_, and PO_4_ centers are depicted by blue, orange, and green octahedra and tetrahedra, respectively, with metal atoms (W and Mo) and heteroatom P located at the center and O atoms located at the apex of polyhedra. Conditions: (i) CH_3_CN, TBABr overnight; (ii) [Pd(PPh_3_)_2_Cl_2_], Et_3_N, DMF, CuI (when required) overnight.

As molecular metal oxides, polyoxometalates (POMs) have astonishing redox properties, which are highly tunable according to their molecular structure, the nature of the metal addenda,^1−4^ the heteroatom,^5^ the countercations,^6,7^ etc. These redox properties have been harnessed in several fields of applications, ranging from (opto)electronic devices^8−10^ to molecular batteries.^11,12^ In this context, vanadates^13,14^ are especially attractive because of the accessibility of mixed-valence species^15^ and, to a lesser extent, molybdates.^16−18^ However, molybdates suffer from higher kinetic lability compared to tungstates.^19,20^ This is heightened in the case of vacant species, used as precursors for the preparation of organic–inorganic POM-based hybrids. Whereas POM hybrids of the PW_11_ and P_2_W_17_ anions are well represented,^21−23^ hybrids based on the PMo_11_ scaffold are scarce.^24,25^ In our previous studies, we have shown the positive effect of Mo when associated with a photosensitizer.^26,27^ We have also described immobilization of the diazonium-terminated hybrids [PM_11_O_39_{Sn(C_6_H_4_)C≡C(C_6_H_4_)N_2_^+^}]^3–^ onto hydrogenated Si, and we have demonstrated the effect of the nature of the metal addenda, Mo versus W, on the electron-transport properties of the resulting molecular junctions.^28,29^ Mixed Mo/W heteropolyanions^30,31^ combine the robustness of tungstates with the ease of reduction of molybdates. Furthermore, when the sites accommodating Mo versus W are precisely defined, it offers an original opportunity to play with localized (Mo^V^) and delocalized (W^V^) spins upon reduction, to potentially design electrically addressable qubits or quantum gates.^32^ In line with our previous work, we have thus chosen monovacant [α-PW_9_Mo_2_O_39_]^7–^ to enlarge the family of Keggin-type POM hybrids. In this contribution, we thus report on the synthesis and characterization of the Sn derivative (TBA)_4_[PW_9_Mo_2_O_39_{Sn(C_6_H_4_I)}] (**K**^**W9Mo2**^_**Sn**_) as a new platform to be subsequently engaged in postfunctionalization reactions (TBA stands for a tetra-*n*-butylammonium cation). A remote ferrocenyl (Fc) unit has also been introduced to give (TBA)_4_[PW_9_Mo_2_O_39_{Sn(C_6_H_4_)C≡C(C_6_H_4_)Fc}] (**K**^**W9Mo2**^_**Sn**_**[Fc]**) in order to provide an internal redox reference for the study of the electrochemical behavior of the new mixed-POM hybrid. The synthetic routes are presented in Scheme 1. In line with our previous studies^28,29,33−36^ and to enlarge the family of available precursors for POM processing onto surfaces, we have also prepared the diazonium-terminated mixed hybrid (TBA)_3_[PW_9_Mo_2_O_39_{Sn(C_6_H_4_)C≡C(C_6_H_4_)N_2_^+^] (**K**^**W9Mo2**^_**Sn**_**[N**_**2**_^**+**^**]**) by the deprotection of (TBA)_4_[PW_9_Mo_2_O_39_{Sn(C_6_H_4_)C≡C(C_6_H_4_)N_3_Et_2_}] (**K**^**W9Mo2**^_**Sn**_**[N**_**3**_**Et**_**2**_**]**). However, its description exceeds the scope of this paper and will be reported elsewhere. The electronic structures of the one- and two-electron-reduced states of **K**^**W9Mo2**^_**Sn**_ (**I** and **II**) have been unraveled through combined spectroelectrochemistry, electron spin resonance (ESR) investigation, and theoretical calculations.

## Results and Discussion

### Synthesis of Mixed-POM Hybrids Derived from K_7_[α-PW_9_Mo_2_O_39_]

While mono- or multivacant POMs have been extensively exploited for the incorporation of extra transition-metal cations (i.e., other than W, Mo, or V) or the preparation of organic–inorganic hybrids, to the best of our knowledge, transition-metal derivatives of K_7_[α-PW_9_Mo_2_O_39_] or the corresponding K_8_[α-SiW_9_Mo_2_O_39_]^37^ have not been reported. During the final writing of this paper, the synthesis and characterization of the Dawson-type organophosphonate hybrid K_6_[P_2_W_15_Mo_2_O_61_(POC_6_H_5_)_2_] has been described, emphasizing the interest in well-defined and not randomly distributed mixed Mo/W POMs.^38^

#### Synthesis of K_7_[α-PW_9_Mo_2_O_39_]

The synthesis of the monovacant K_7_[α-PW_9_Mo_2_O_39_] was first reported in 1977,^39^ starting from the sodium salt of [HPW_9_O_34_]^8–^. Note that the latter was initially assigned as a B,β-isomer, but lately has been corrected as a A,β-isomer.^40^ Some years ago, some of us have thus reported the synthesis of K_7_[α-PW_9_Mo_2_O_39_] by the reaction between K_9_[A,α-PW_9_O_34_] and sodium molybdate.^41^ As usual, in the POM synthesis, the pH of the solution should be carefully controlled and kept between 4.5 and 5. In spite of these precautions and many attempts, the ^31^P NMR spectrum of K_7_[α-PW_9_Mo_2_O_39_] displays a small impurity at −10.28 ppm close to the main signal at −9.67 ppm. The degree of purity was estimated as 97%. This confirms the existence of a predominant isomer, assigned to two adjacent corner-shared MoO_6_ octahedra, or the 1,2-isomer according to IUPAC numbering of the metal atom positions.^42^

#### Functionalization of K_7_[α-PW_9_Mo_2_O_39_]: Synthesis of the **K**^**W9Mo2**^_**Sn**_ Platform

The synthesis of **K**^**W9Mo2**^_**Sn**_ follows those of (TBA)_4_[PW_11_O_39_{Sn(C_6_H_4_I)}] (**K**^**W**^_**Sn**_) and (TBA)_4_[PMo_11_O_39_{Sn(C_6_H_4_I)}] (**K**^**Mo**^_**Sn**_)^43,44^ by the reaction between the monovacant POM and the trichloroorganotin derivative [Cl_3_Sn(C_6_H_4_I)]. However, the synthesis of POM hybrids is rarely straightforward, and some adaptations are required. **K**^**W**^_**Sn**_ is prepared by the reaction between K_7–*x*_Na_*x*_[α-PW_11_O_39_] and [Cl_3_Sn(C_6_H_4_I)] in water under pH control, followed by precipitation by the addition of tetrabutylammonium bromide (TBABr). Direct extension to K_7_[α-PW_9_Mo_2_O_39_] only gave mixtures of compounds according to ^31^P NMR spectroscopy. Because **K**^**Mo**^_**Sn**_ is prepared in acetonitrile (CH_3_CN) from the reaction between (TBA)_4_H_3_[α-PMo_11_O_39_] and [Cl_3_Sn(C_6_H_4_I)] in the presence of TBABr and triethylamine (NEt_3_) used to neutralize the release of hydrochloric acid (HCl), we also tried to start from a suspension of K_7_[α-PW_9_Mo_2_O_39_] in CH_3_CN in the presence of [Cl_3_Sn(C_6_H_4_I)], NEt_3_, andTBABr, with the latter being used as a transfer agent. This procedure also led to a mixture of compounds. Finally, we found that the presence of NEt_3_ was not required if the amount of water contained in CH_3_CN was limited by prior distillation onto calcium hydride (CaH_2_). After filtration of some unreacted K_7_[α-PW_9_Mo_2_O_39_], the solvent was evaporated, and the resulting oil was dissolved in dichloromethane (DCM) in the presence of TBABr. Subsequent workup included washing of the organic phase with water to eliminate mineral salts, evaporation of the solvent, redissolution in CH_3_CN, and final precipitation by the addition of ethanol to recover **K**^**W9Mo2**^_**Sn**_ as a white solid (yield 46%).

#### Postfunctionalization of the **K**^**W9Mo2**^_**Sn**_ Platform: Synthesis of **K**^**W9Mo2**^_**Sn**_**[Fc]**

Once functionalized, the **K**^**W**^_**Sn**_, **K**^**Mo**^_**Sn**_, and now the current **K**^**W9Mo2**^_**Sn**_ platforms are robust enough to be engaged in postfunctionalization reactions performed in organic solvents. Pd-catalyzed C–C cross-coupling reactions offer an unlimited number of possibilities to anchor a remote functional group for further integration of POM hybrids into advanced architectures or processing into molecular materials.^22,45^ In this contribution, we have chosen to illustrate Sonogashira-type reactions between **K**^**W9Mo2**^_**Sn**_ and alkynes functionalized by a Fc unit, subsequently used as an electrochemical probe, and, as a second example, by a diethyltriazene group featuring a protected diazonium function. The reaction between **K**^**W9Mo2**^_**Sn**_ and an excess of the appropriate alkyne was carried out in anhydrous *N*,*N*-dimethylformamide (DMF) in the presence of distilled NEt_3_ and the *cis*-[PdCl_2_(PPh_3_)_2_] catalyst (plus, if needed, CuI). After the elimination of nonsoluble materials, the crude compounds were precipitated by the addition of diethyl ether, redissolved in CH_3_CN, in the presence of TBABr, and recovered as pure products by the addition of ethanol (yield 77.7%).

### Characterization by IR and NMR Spectroscopies

All compounds have been characterized by elemental analysis, mass spectrometry (MS), and IR and ^1^H and ^31^P NMR spectroscopies. The ^1^H and ^31^P NMR spectra together with the electrospray ionization MS (ESI-MS) spectra are presented in the Supporting Information (SI). Integration of the signals corresponding to the aromatic protons relative to the signals characteristic of the TBA^+^ cations is a good indicator of the accuracy of the proposed molecular formula, which is further confirmed by the molecular peak observed for the POM framework part in the ESI-MS spectra. The purity of the compounds was further attested by ^31^P NMR. Upon going from K_7_[α-PW_9_Mo_2_O_39_] to **K**^**W9Mo2**^_**Sn**_, the ^31^P chemical shift experienced a small upfield shift from −9.67 to −9.81 ppm, in agreement with completion of the POM vacancy. Note, however, that the corresponding spectra were recorded in different solvents, LiCl/D_2_O versus CD_3_CN, which precludes any deeper comparison. In the **K**^**W12-xMox**^ series, the ^31^P chemical shift δ_P_ was found to vary linearly with *x*: the higher *x* is, the higher δ_P_ is.^39^ This is also verified here: δ_P_ for **K**^**W9Mo2**^_**Sn**_ (−9.81 ppm) lying between δ_P_ of **K**^**W11**^_**Sn**_ (−10.76 ppm) and **K**^**Mo11**^_**Sn**_ (−2.15 ppm). As expected, and previously observed for the full tungstate or molybdate homologues, the value of the ^31^P chemical shift associated with **K**^**W9Mo2**^_**Sn**_ is barely altered after postfunctionalization, with a value of −9.85 ppm associated with **K**^**W9Mo2**^_**Sn**_**[Fc]**.

**Figure 1:**
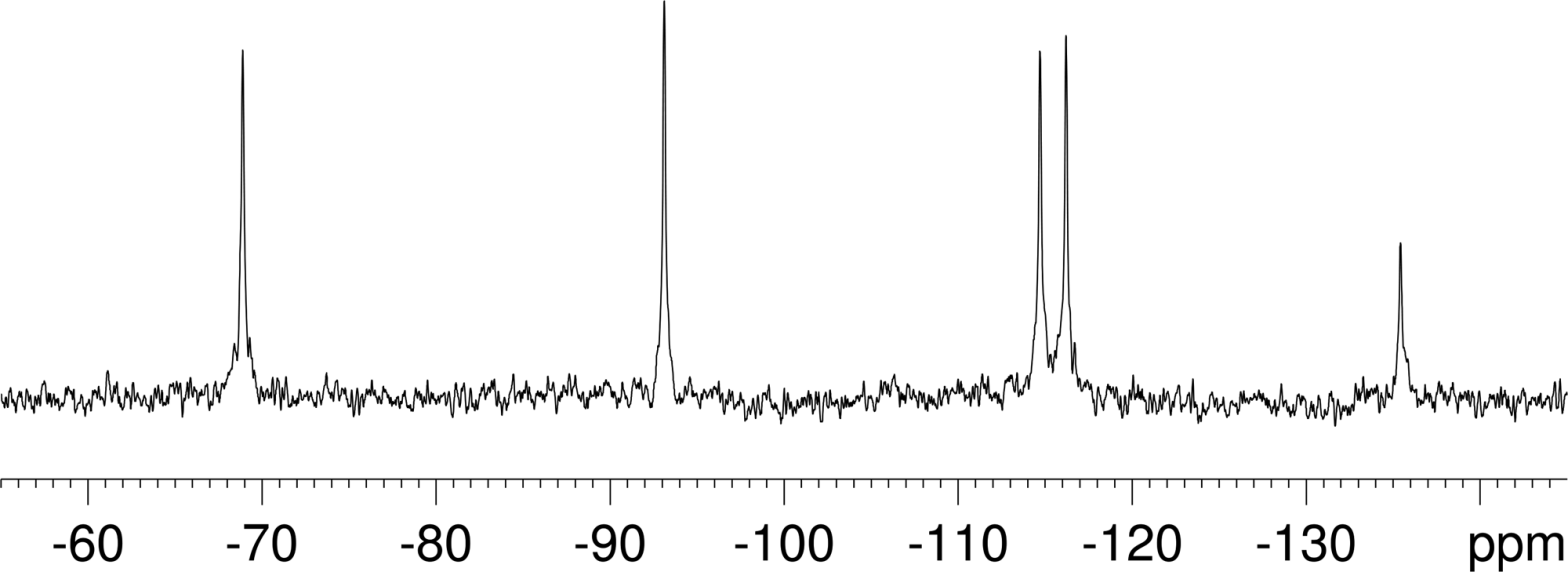
^183^W NMR spectrum of **K**^**W9Mo2**^_**Sn**_ in CH_3_CN.

The ^183^W NMR spectrum of **K**^**W9Mo2**^_**Sn**_ is compliant with *C*_*s*_ symmetry with five resonance lines at −69.0, −93.4, −114.7, −116.2, and −135.5 ppm of relative intensities of 2:2:2:2:1 (Figure 1). It is very close to the spectrum reported for K_5_[α-SiW_9_Mo_2_VO_40_] (−90, −93.4, −105.3, −109.4, and −134.8 ppm), except that the most downfield signal that was attributed to the two W centers sharing edges with V was much broader.^46^ This further supports retention of the A-αPW_9_, structure with two corner-shared MoO_6_ octahedra and little, if any isomerization (also supported by the ^31^P NMR spectrum).

The IR spectrum of **K**^**W9Mo2**^_**Sn**_ displays the characteristic signature of a Keggin structure with three strong bands at 960, 885, and 808 cm^–1^, corresponding to ν_as_(M–O_d_), ν_as_(M–O_b_–M), and ν_as_(M–O_c_–M), respectively.^47^ These values are very close to those of **K**^**W11**^_**Sn**_ (963, 885, and 814 cm^–1^)^37^ and higher than those of **K**^**Mo11**^_**Sn**_ (943, 866, 806, and 785 cm^–1^).^44^ They thus reflect both the composition of the polyanion and its total charge, with the vibrations of tungstates occurring at higher wavenumbers than those of molybdates and shifted to lower values with an increase of the total charge.^47^ The presence of the phosphate group is disclosed by the ν_as_(P–O) vibration at 1070 cm^–1^, and additional bands at 2962 (m), 2937 (m), 2873 (m), 1481 (m), and 1379 (w) cm^–1^ correspond to the stretching and bending modes of CH_*n*_ of the tetrabutylammonium cations. The stretching bands of the C=C bonds of the aromatic ring, expected in the 1500–1600 cm^–1^ region, are too weak to be assigned precisely. Finally, at low wavenumbers, in the 400–300 cm^–1^ range, the two-band patterns at about 380 cm^–1^ (sharp strong) and 330 cm^–1^ (sharp medium) are characteristic of the α-isomer.^48^ Postfunctionalization has little effect on the vibrations of the metal oxide scaffold.

The antisymmetric stretching ν(P–O) mode of the central PO_4_^3–^ group deserves a special comment. It is very sensitive to the symmetry of the anion: while TBA_3_[PW_12_O_40_] and TBA_3_[PMo_12_O_40_] display single bands at 1080 and 1063 cm^–1^, respectively,^47^ the monovacant species TBA_4_H_3_[PW_11_O_39_] is characterized by a splitting of the previous band into two components at 1110 and 1060 cm^–1^ separated by 50 cm^–1^ (1079 and 1052 cm^–1^ for TBA_4_H_3_[PMo_11_O_39_] and 1087 and 1050 cm^–1^ for K_7_[α-PW_9_Mo_2_O_39_]).^24^ In metal-substituted [PW_11_O_39_ML]^(7–*n*)–^, the splitting between the two ν(P–O) vibrations is reduced and its variation is indicative of the interaction of the added ML^*n*+^ cation with the vacant POM and central PO_4_^3–^ group: the lower the splitting, the better the cation refills the vacancy and restores a pseudotetrahedral geometry like in the complete POMs (L = ligand).^49^ This has been reported for the incorporation of first-row transition-metal cations, and we have also observed it in the metal nitrido derivatives [PW_11_O_39_MN]^(10–*n*)–^ (M = Ru^VI^, Cr^V^).^50,51^ The ionic radius of the extra cation compared to that of W^VI^/Mo^VI^ and its electronic configuration are likely relevant parameters, but the composition of the POM scaffold also appears to play a role: the IR spectra of **K**^**W**^_**Sn**_ and **K**^**W9Mo2**^_**Sn**_ display a single ν(P–O) band at 1070 cm^–1^ (but a shoulder at 1055 cm^–1^ is present on the IR spectra of the postfunctionnalized species **K**^**W9Mo2**^_**Sn**_**[Fc]**, whereas the ν(P–O) band is clearly split into two components at 1062 and 1035 cm^–1^ for **K**^**Mo**^_**Sn**_ (Figures S5, S8, and S11).^44^ This suggests a decrease of the distortion from **K**^**Mo**^_**Sn**_ to **K**^**W9Mo2**^_**Sn**_ and an increase of the robustness of the Sn insertion to what we are looking for.

### Electrochemical Characterization

**Figure 2:**
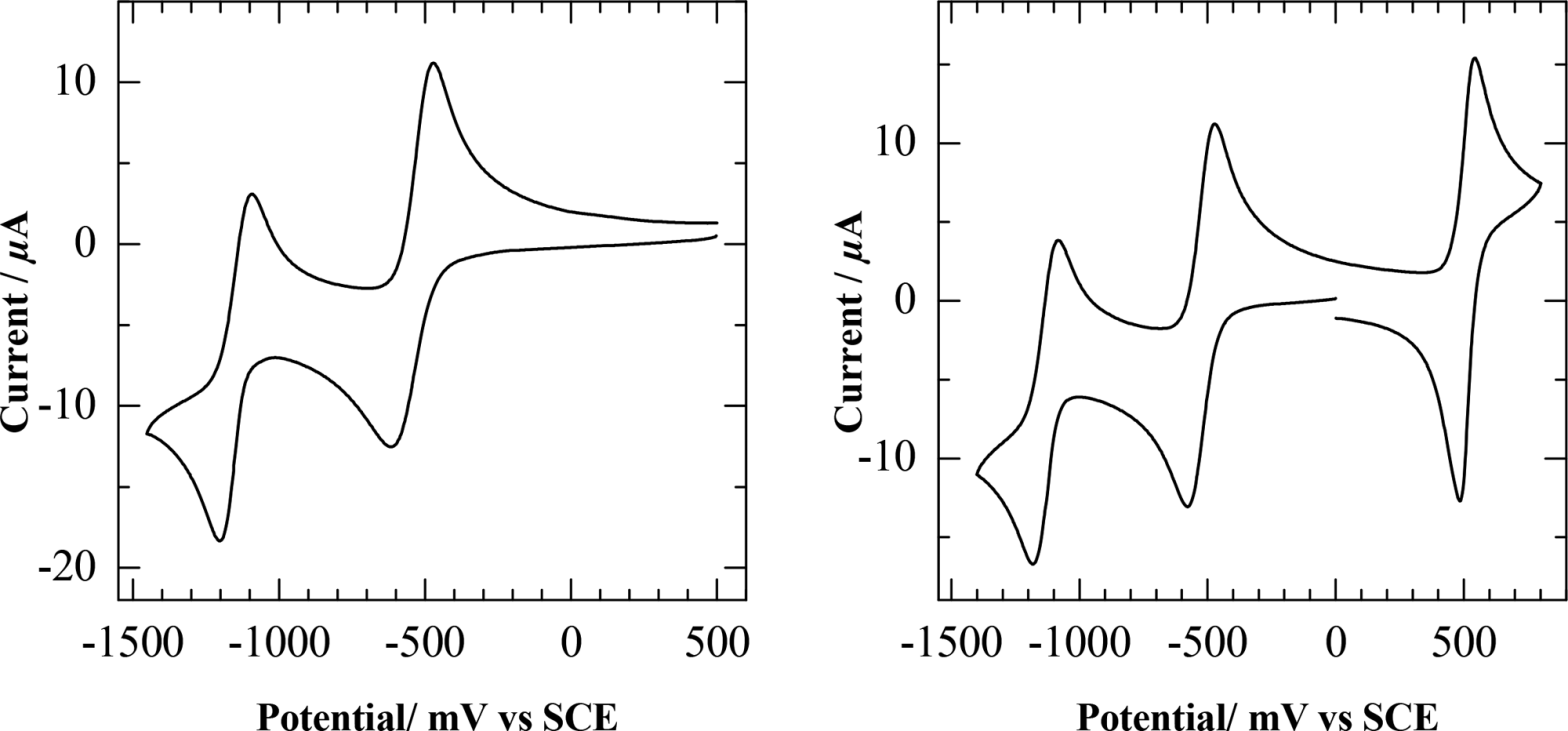
Cyclic voltammograms of 0.1 mM **K**^**W9Mo2**^_**Sn**_ (left) and **K**^**W9Mo2**^_**Sn**_**[Fc]** (right) in CH_3_CN (0.1 M TBAPF_6_) at a glassy carbon electrode. Potentials are given versus a SCE electrode; scan rate = 0.1 V s^–1^.

The cyclic voltammograms of **K**^**W9Mo2**^_**Sn**_ and **K**^**W9Mo2**^_**Sn**_**[Fc]** have been recorded in CH_3_CN at a glassy carbon electrode. They are depicted in Figure 2, and relevant electrochemical data for these POM hybrids and others are summarized in Table 1. Both display two reduction processes around −0.55 and −1.15 V versus saturated calomel electrode (SCE) assigned to the POM framework and, for **K**^**W9Mo2**^_**Sn**_**[Fc]**, an oxidation process at +0.51 V versus SCE (Ep_a_ = +0.54, Ep_c_ = +0.48, and ΔEp = 0.06 V) attributed to the ferrocene unit. **Fc** was used as an internal redox reference and allows a direct comparison of the intensities of the waves of **K**^**W9Mo2**^_**Sn**_**[Fc]** because a unique diffusion coefficient is to be considered for the two covalently connected redox-active units, **K**^**W9Mo2**^_**Sn**_ and **Fc**. It thus becomes clear that all processes correspond to monoelectronic transfer. The reduction potentials of **K**^**W9Mo2**^_**Sn**_ and **K**^**W9Mo2**^_**Sn**_**[Fc]** are very similar and in the range of experimental uncertainty. As we have previously noted for organotin (and organosilyl) derivatives, postfunctionalization has almost no effect on the redox potentials.^22,52−54^ This suggests an electronic decoupling between the POM core and the organic tether, at variance with what was reported on organophosphonate POM hybrids.^55,56^

**Table 1:** Electrochemical Data: Cathodic Peak Potential Ep_c_, Anodic Peak Potential Ep_a_, Peak-to-Peak Separation ΔEp, Cathodic-to-Anodic Peak Intensity Ratio ip_c_/ip_a_, and Midpoint Potential Ep_1/2_

POM	Ep_c_	Ep_a_	ΔEp	ip_c_/ip_a_	Ep1/2	Ep_c_	Ep_a_	ΔEp	ip_c_/ip_a_	Ep1/2	ref
**K**^**W**^_**Sn**_					–0.99					–1.46	(27)
**K**^**Mo**^_**Sn**_					–0.50					–0.91	(46)
**K**^**W9Mo2**^_**Sn**_	–0.62	–0.47	0.15	0.97	–0.55	–1.20	–1.09	0.11	1.13	–1.15	
**K**^**W9Mo2**^_**Sn**_**[Fc]**	–0.58	–0.47	0.11	1.04	–0.53	–1.19	–1.09	0.10	1.08	–1.14	

The reduction of molybdates is known to be easier than the reduction of tungstates,^31,57−59^ and indeed the reduction potentials of **K**^**Mo**^_**Sn**_ are shifted toward less negative values compared to those of the **K**^**W**^_**Sn**_ homologue (Table 1). In addition, the first reduction potentials of **K**^**Mo**^_**Sn**_ and **K**^**W9Mo2**^_**Sn**_ are very close to each other. We can thus draw the conclusion that the first reduction process of **K**^**W9Mo2**^_**Sn**_ and of the related postfunctionalized species leads to the reduction of one of the two Mo centers. Assignment of the second reduction event is not straightforward: transfer of a second electron on the second Mo could be energy-favored but at the expense of a strong electrostatic repulsion. Conversely, the reduction of one W will bring additional delocalization on the tungstate scaffold. Note also that the difference between both reduction potentials is larger in the heterometallic **K**^**W9Mo2**^_**Sn**_ (0.60 V) than in the homometallic **K**^**W**^_**Sn**_ (0.46 V) and **K**^**Mo**^_**Sn**_ (0.41 V) species, which could denote either a second electron transfer to tungsten or a strong repulsion between two adjacent Mo^V^. This issue was further addressed both theoretically and experimentally through the electrochemical preparation of the one- and two-electron-reduced species **1e-K**^**W9Mo2**^_**Sn**_ (**I**) and **2e-K**^**W9Mo2**^_**Sn**_ (**II**) and their characterization via UV–vis and ESR spectroscopies. This will be discussed in a following part.

### Spectroscopic Insights into the Electronic Structure of the One- and Two-Electron-Reduced **K**^**W9Mo2**^_**Sn**_ (**I** and **II**)

#### Spectroelectrochemistry–UV–Vis Spectra of the Reduced Forms of **K**^**W9Mo2**^_**Sn**_

**Figure 3:**
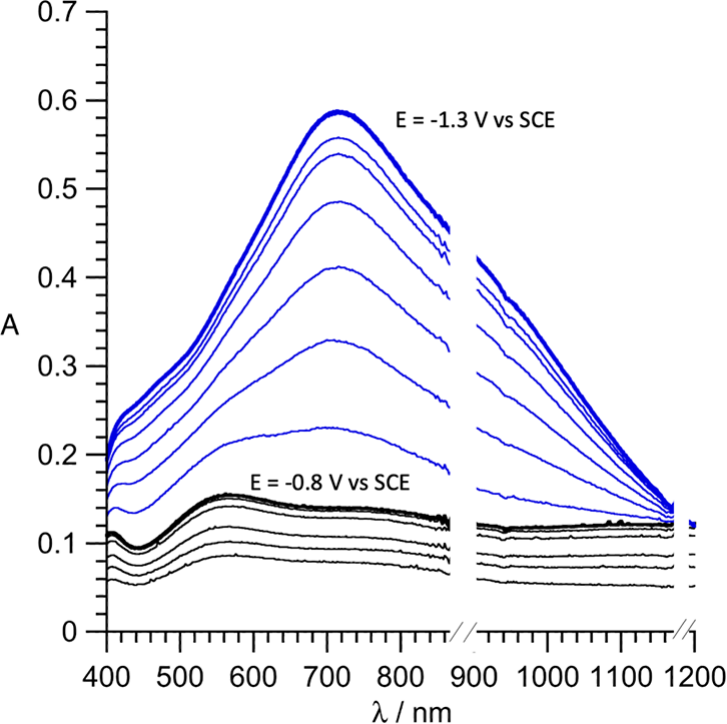
Absorbance (*A*) monitoring of the electronic spectrum of a 0.2 mM solution of **K**^**W9Mo2**^_**Sn**_ in CH_3_CN (0.1 M TBAPF_6_), upon application of a constant potential of −0.8 V versus SCE to generate **I** (black) and then −1.3 V versus SCE to generate **II** (blue).

The stepwise reduction of a 0.2 mM solution of **K**^**W9Mo2**^_**Sn**_ in CH_3_CN (0.1 M TBAPF_6_) has been monitored by spectroelectrochemistry (Figure 3). Applying a potential of −0.8 V versus SCE induced the growth of a broad band around 560 nm (ε = 750 M^–1^ cm^–1^), which is compliant with the absorption spectra of 1e-[SiW_11_Mo^V^O_40_]^5–^ (λ = 510 nm; ε = 950 M^–1^ cm^–1^),^60^ 1e-[PW_11_Mo^V^O_40_]^4–^ (λ = 500 nm; ε = 1150 M^–1^ cm^–1^), and 5e-[H_x_SiW_9_Mo^V^V^III^_2_O_40_]^11–x^ (λ = 488 nm; ε = 785 M^–1^ cm^–1^).^61^ At this step, the solution of **I** was more violet than blue, like the solution of 1e-[PW_11_Mo^V^O_40_]^4–^, which is reported to be red-violet. This broad absorption has been variously attributed to a Mo^V^-centered d–d transition^60^ or heteronuclear Mo^V^ → W^VI^ intervalence charge transfer (IVCT)^37,61^ and might simply conceal a superimposition of several electronic absorptions.

Decreasing the applied potential to −1.3 V versus SCE strengthened a contribution around 720 nm (ε = 2950 M^–1^ cm^–1^) similar to the main contribution observed in the spectra of 1e-[β-SiW_9_Mo_3_O_40_]^5–^ and 1e-[SiMo_12_O_40_]^5–^ or 2e-[PMo_12_O_40_]^5–^ (related to the reduction of Mo)^60,62,63^ but also of 1e-[SiW_12_O_40_]^5–^ and 1e-[PW_12_O_40_]^4–^ (related to the reduction of W).^64^ It underscores that the absorption maxima of such broad bands are not very specific. 2e-[SiW_12_O_40_]^6–^ and 2e-[PW_12_O_40_]^5–^ are rather characterized by an absorption at lower wavelength (λ = 625 and 653 nm, respectively).^64^ In these examples, homonuclear IVCT, Mo^V^ → Mo^VI^ or W^V^ → W^VI^, is possible. Solutions of **II** are blue.

The global shift of the absorption spectra to higher wavelength/lower energy upon the successive reduction steps of **K**^**W9Mo2**^_**Sn**_ is unusual and at variance with the trend indeed observed along the reduction of [α-SiW_10_Mo_2_O_40_]^4–^: 1e-[ α-SiW_10_Mo_2_O_40_]^5–^ and 2e-[α-SiW_10_Mo^V^_2_O_40_]^6–^ are characterized by an absorption at λ = 590 nm (ε = 930 M^–1^ cm^–1^) and λ = 500 nm (ε = 3300 M^–1^ cm^–1^), respectively (the two Mo atoms are proposed to belong to the same triad in this example, sharing edges and not corners like in our case).^65^ Similarly, 3e-[SiW_9_Mo^V^_2_V^IV^O_40_]^7–^ is characterized by an absorption at λ = 490 nm (the rather large ε = 4070 M^–1^ cm^–1^ value in this case is due to an additive contribution of V^IV^).^37^ Taken altogether, these experimental observations would suggest association of the second reduction event in **K**^**W9Mo2**^_**Sn**_ to W (**II**_**MoW**_) rather than to the second Mo (**II**_**MoMo**_), with an unexpected effect of the break of symmetry introduced by the Sn functionalization. However, UV–vis spectra of the reduced POMs are finally close to one another, be it in the Keggin or Dawson series,^55,64,66,67^ with large bands resulting from the overlap of multiple absorption events, d–d, and homo- and heteronuclear IVCT transitions, occurring in the whole visible–near-IR range, and with relative intensities sensitive to structural isomerism.^65^ At this stage, drawing a definitive conclusion regarding the location of the second electron in **II**, on W (**II**_**MoW**_) or on Mo (**II**_**MoMo**_), is thus gambling. Further insights will be provided by theoretical calculations.

#### ESR Spectroscopy of the Reduced Forms of [PW_9_Mo_2_O_39_{Sn(C_6_H_4_I)]^4–^, 1e-[PW_9_Mo_2_O_39_{Sn(C_6_H_4_I)]^5–^ (**I**), and 2e-[PW_9_Mo_2_O_39_{Sn(C_6_H_4_I)]^6–^ (**II**, Either **II**_**MoW**_ or **II**_**MoMo**_)

Whatever the location of the second electron in **II**, the final spin state, *S* = 0 or *S* = 1, is to be determined. Provided electron delocalization, a singlet state is generally observed for homometallic POM reduced to an even number of electrons.^68−70^ In mixed POMs, the picture is to be nuanced.^2^ The properties of tungstovanadates have been especially investigated, whereas discussing the electronic structure of reduced [PW_10_V_2_O_40_]^5–^ or [PMo_10_V_2_O_40_]^5–^ is meaningless because the general formula hides mixtures of five geometrical isomers, [SiW_10_V_2_O_40_]^6–^, yet corresponds to a single 1,2-isomer with adjacent corner-shared VO_6_ octahedra.^42,71^ The ESR spectrum of 1e-[SiW_10_V_2_O_40_]^7–^ displays a 15-line ESR pattern, in agreement with an electron delocalized over the two V atoms (*I* = ^7^/_2_), but magnetic susceptibility measurements carried out on 2e-[SiW_10_V_2_O_40_]^8–^ revealed that there is essentially no magnetic exchange between the two V^IV^ ions, thus two isolated spins ^1^/_2_.^72^ This is in contrast with the behavior of [SiW_9_V_3_O_40_]^7–^, with one electron trapped on one V atom in 1e-[SiW_9_ V_3_O_40_]^7–^ and two antiferromagnetically coupled electrons in 2e-[SiW_9_V_3_O_40_]^8–^ (*J* = −34.9 cm^–1^). The striking difference has been ascribed to some variation of the V–O–V angle values, possibly modulated by protonation.

**Figure 4:**
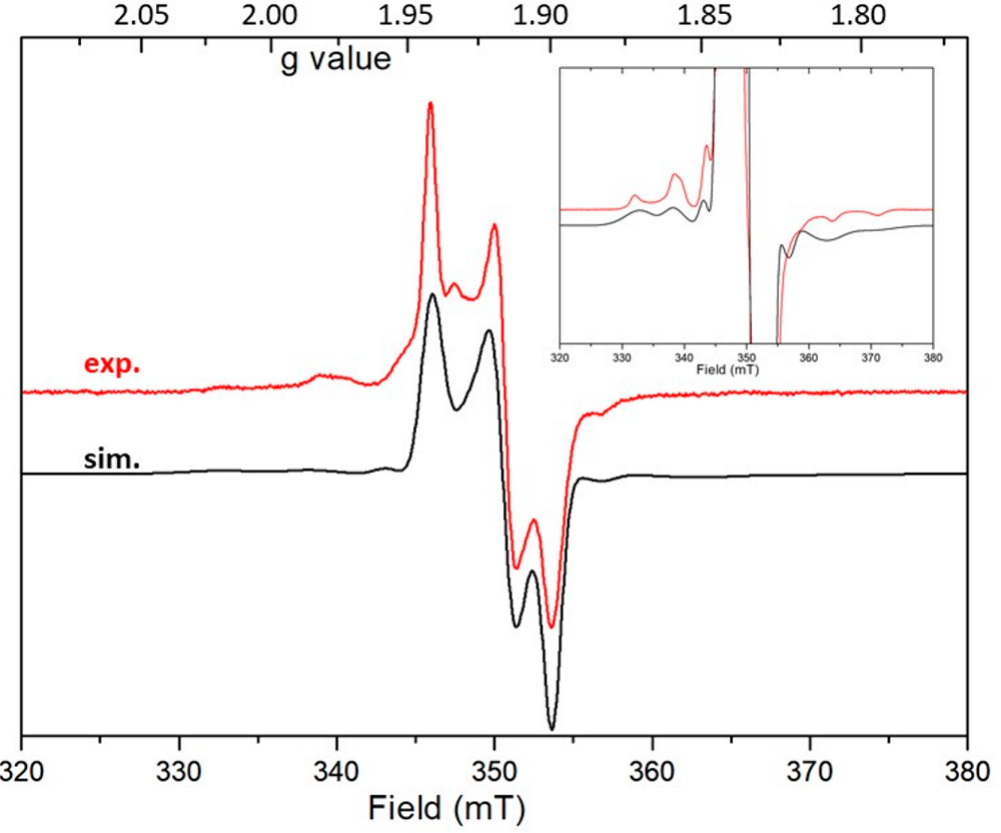
X-band ESR spectrum of a frozen solution (20 K) of [PW_9_Mo_2_O_39_{Sn(C_6_H_4_I)}]^5–^ obtained by spectroelectrochemistry (solution in CH_3_CN and 0.1 M TBAPF_6_). Inset: Amplification of the central part unveiling the hyperfine coupling.

During the spectroelectrochemical study on [PW_9_Mo_2_O_39_{Sn(C_6_H_4_I)}]^4–^, aliquots of the solution have been frozen (20 K) to be probed by ESR spectroscopy. Parallel to the growth of the first UV–vis feature, one can clearly observe in X-band ESR the apparition of a quite rhombic signature around *g* = 2 (Figure 4). Interestingly enough, one can clearly observe on the side of this main feature hyperfine lines that could account for about 25% of the Mo atoms bearing a ^5^/_2_ nuclear spin [^95^Mo (15.7%) and ^97^Mo (9.6%)]. Indeed, satisfactory simulation of these ESR features was obtained with *Easy Spin* using the naturally abundant isotopes of Mo with a rhombic **g** matrix (*g*_*x*_ = 1.941, *g*_*y*_ = 1.916, and *g*_*z*_ = 1.899) and *A*_*x*_ = 135 MHz (∼50 G) and *A*_*z*_ = 180 MHz (∼68 G) (*A*_*y*_ could not be resolved).^73^ These values are quite in line with those obtained by Pope for the monoreduced [PMo^V^W_11_O_40_] anion with an axial spin system (*g*_∥_ = 1.918, *A*_∥_ = 81.3, *g*_⊥_ = 1.937, and *A*_⊥_ = 33.2 G)^61,74^ and confirm that the first reduction is localized on a Mo atom in **I**. Upon further reduction, a progressive decrease of the signal is observed with no new features grown, indicating that the two-electron-reduced species **II** is silent in these conditions.

The amount of **II** obtained by spectroelectrochemistry was too low to allow magnetic susceptibility measurements. The chemical reduction path has also been investigated, using sodium naphthalenide as the reducing agent.^75^ However, we have been faced with the (expected) high sensitivity of the reduced species to reoxidation, so that only characterization of **I** could be carried out (for electronic and ESR spectroscopies, see the SI, corroborating the previously described features).

### Theoretical Calculations

**Figure 5:**
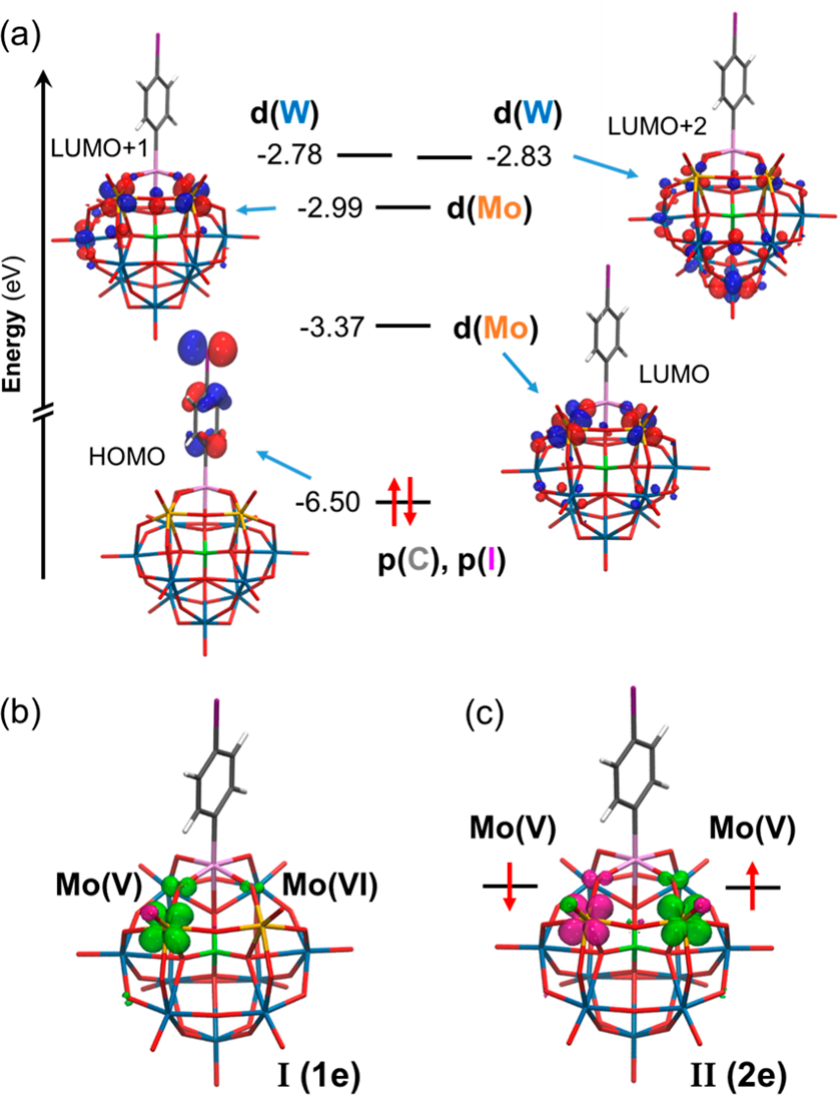
(a) Schematic frontier molecular orbital diagram for **K**^**W9Mo2**^_**Sn**_ at the B3LYP-D3 level. Table S1 compiles the frontier molecular orbital energies for **K**^**W9Mo2**^_**Sn**_ calculated with different density functionals. (b and c) Spin-density distributions for the one- and two-electron-reduced species **I** and **II**, respectively, showing successive reduction steps of the Mo centers and the antiferromagnetic nature of **II**. Color code: W, blue; Mo, orange; Sn, pink; P, green; I, purple; C, gray; O, red; H, white.

To better understand the electronic structure of **K**^**W9Mo2**^_**Sn**_ and the localization of electrons in its one- and two-electron-reduced counterparts, we next conducted DFT calculations. As shown in Figure 5a, the HOMO of the fully oxidized system is localized on the organic moiety, while the LUMO and LUMO+1 are both centered on Mo^VI^ ions, accounting for antibonding combinations of Mo d and O p orbitals. Notably, the first empty molecular orbitals with the main contribution from W d orbitals are the quasi-degenerated LUMO+2 and LUMO+3, which lie ca. 0.20 eV above the LUMO+1. The first reduction of **K**^**W9Mo2**^_**Sn**_ was thus found to occur in one of the Mo centers (see Figure 5b for the spin-density representation), as was already inferred from electrochemical studies (*vide supra*). Most importantly, DFT calculations revealed that the intriguing second reduction is also Mo-centered, as shown in the spin-density distribution of **II** (Figure 5c). Supporting this assignment, the DFT-calculated reduction potentials for the two successive Mo^VI^ → Mo^V^ reduction steps (−0.54 and −1.21 V vs SCE at the B3LYP-D3 level) are in rather good agreement with the experimental values (Table 2). In fact, using a model system whereby one of the Mo centers was replaced by W, we estimated the potential for a W-centered second reduction to be excessively negative (−1.74 V vs SCE) to match the experimental value of −1.14 V. Similar results for Mo^VI^ → Mo^V^ reduction steps were found using ωB97X-D and HSE06 functionals (Table 2).

**Table 2:** Electronic and Energetic Parameters for Calculated **K**^**W9Mo2**^_**Sn**_ and Its Reduced Partners with Different Density Functionals

	*E*°_red_^a^			spin-density distribution in **II**^c^		
functional	0e/1e	1e/2e	Δ*E*°_red_^b^	Mo1	Mo2	Δ*G*(triplet–singlet)^d^
B3LYP-D3	–0.54	–1.21	0.67	–0.80	0.79	+0.8
ωB97X-D	–0.43	–1.16	0.73	–0.84	0.85	+0.3
HSE06	–0.55	–1.30	0.75	–0.82	0.82	+0.6
exp.	–0.55	–1.15	0.61			

a Redox potentials (V) were calculated against SCE, taking an absolute potential of 4.67 V for SCE in nonaqueous conditions.^78^

b Computed redox potential difference between the first and second electron reductions.

c Mulliken spin densities for the singlet (broken-symmetry) state.

d Energy difference (kcal mol^–1^) between triplet and singlet states computed at different DFT levels.

In agreement with the ESR measurements, our DFT calculations predict the ground state of **II** to be an open-shell singlet, whereby the two “extra” electrons are unpaired but antiferromagnetically coupled (Figure 5c and Table 2). The open-shell singlet solution obtained with the broken-symmetry formalism lies 0.8 kcal mol^–1^ below in energy than the most stable triplet state and 6.8 kcal mol^–1^ below the closed-shell singlet, in which both electrons are localized in a molecular orbital with the contribution from d-type orbitals of both Mo centers. This is in line with previous theoretical studies on the diamagnetism of bireduced POMs, which showed that the hopping integrals between neighboring centers and the electron–electron repulsion stabilize the singlet over the triplet state.^70,76,77^ However, it is important to note that the singlet-state stabilization mechanism in systems with localized electrons differs from those involving hopping electrons. Similar behavior was found using the ωB97X-D and HSE06 functionals. Spin-density values close to that for Mo atoms (positive and negative signs for α and β electrons, respectively) are consistent with the presence of Mo^V^ ions. The rest of the spin density is delocalized over the oxo ligands of the Mo^V^ centers, as shown in Figure 5c. Finally, the evolution of the DFT-simulated absorption spectra upon going from **I** to **II** (see Figure S10 and related text) reproduces rather well the experimental variations observed in Figure 3, further supporting the herein-described electronic structure for species **II**.

## Concluding Remarks

The family of Keggin-type POM hybrids has been enlarged with mixed Mo/W species, **K**^**W9Mo2**^_**Sn**_, and its Fc derivative. Both species have similar electroactivities, with reduction processes facilitated by the incorporation of Mo. This widens the range of redox potential tuning often harnessed in POM-based molecular materials. The monoreduced species, [PW_9_Mo^VI^Mo^V^O_39_{Sn(C_6_H_4_I)}]^5–^, is stable below −0.55 V versus SCE and characterized by the main electronic transition at 560 nm. According to time-dependent DFT (TD-DFT) calculations, this band is assigned to a superimposed Mo^V^ d–d transition and Mo^V^ → W^VI^ IVCT, while Mo^V^ → Mo^VI^ IVCT lies around 1000 nm. The intensity increase and unusual red shift of the UV–vis spectrum upon further reduction are rather well reproduced by the simulation, in agreement with the apparent maximum found experimentally at 720 nm. Once corroborated by the spectroscopic data, DFT calculations furthermore unveil the site of the second reduction. The bireduced species should thus be described as [PW_9_Mo_2_^V^O_39_{Sn(C_6_H_4_I)}]^6–^, with an open-shell-singlet ground state, consistently silent in ESR. This plural approach, confronting the experimental and theoretical data to the abundant literature dealing with reduced POMs, points out the intricacy of their electronic structure and the difficulty to properly attribute the observed electronic events on the basis of a sole technique. This is illustrated by the UV–vis spectra with broad bands that result from the overlap of so many transitions, metal-centered or metal-to-metal, so that the quoted absorption maxima are finally of low significance.

## Experimental Section

### General Procedures

Chemicals and solvents were obtained from Aldrich or Acros and used as received, except NEt_3_ and CH_3_CN, which were distilled from CaH_2_. K_7_-α[PW_9_Mo_2_O_39_] was prepared as previously described.^39,41^^1^H (400 MHz), ^31^P (121.5 MHz), and ^183^W (25 MHz) NMR spectra were recorded on a Bruker Avance III Nanobay 400 MHz spectrometer equipped with a BBFO probehead (^1^H and ^31^P NMR) or on a Bruker Avance III 600 MHz spectrometer equipped with a BBO probehead (^183^W NMR, 10-mm-o.d. tube). Chemical shifts are quoted as parts per million (ppm) relative to tetramethylsilane using the solvent signals as a secondary standard for ^1^H and relative to 85% H_3_PO_4_ for ^31^P and to a 2 M Na_2_WO_4_ alkaline solution in D_2_O for ^183^W (s, singlet; d, doublet; t, triplet; sex, sextet; m, multiplet), and coupling constants (*J*) are quoted in hertz (Hz). The IR spectrum of the powder was recorded from a KBr pellet on a Jasco FT/IR 4100 spectrometer. Elemental analyses were performed at the Institut de Chimie des Substances Naturelles, Gif sur Yvette, France. High-resolution ESI-MS spectra were recorded using an LTQ Orbitrap hybrid mass spectrometer (Thermofisher Scientific, Bremen, Germany) equipped with an external ESI source operated in the negative-ion mode. Spray conditions included a spray voltage of 3 kV, a capillary temperature maintained at 280 °C, a capillary voltage of −30 V, and a tube lens offset of −90 V. Sample solutions in CH_3_CN (10 pmol μL^–1^) were infused into the ESI source by using a syringe pump at a flow rate of 180 μ h^–1^. MS spectra were acquired with the Orbitrap analyzer with a theoretical mass resolving power (Rp) of 100000 at *m*/*z* 400, after ion accumulation to a target value of 105 and a range detection from *m*/*z* 300 to 2000. All data were acquired using external calibration with a mixture of caffeine, a MRFA peptide, and Ultramark 1600 dissolved in Milli-Q water/HPLC-grade CH_3_CN (50/50, v/v).

Cyclic voltammetry was performed in a three-electrode cell, with a glassy carbon working electrode, a platinum counter electrode, and a saturated Hg_2_Cl_2_/KCl reference electrode fitted with a bridge containing a saturated aqueous LiCl solution.

#### Synthesis of TBA_4_[PW_9_Mo_2_O_39_{Sn(C_6_H_4_I}] (**K**^**W9Mo2**^_**Sn**_)

[Cl_3_SnC_6_H_4_I] (95.5 mg, 0.223 mmol) and an excess of TBABr (310 mg, 0.962 mmol) were placed in 20 mL of dried CH_3_CN. The latest solution was transferred onto K_7_[PW_9_Mo_2_O_39_] (500 mg, 0.132 mmol) and the resulting suspension stirred overnight at room temperature. After centrifugation, the filtered supernatant was concentrated under reduced pressure and led to a greenish oil. This oil was then dissolved into 10 mL of DCM with an excess of TBABr (200 mg, 0.620 mmol) and the solution stirred for 1 h at room temperature. The organic phase was washed by distilled water (3 × 30 mL), and finally it was evaporated under reduced pressure, leading to a white solid. The solid was dissolved in 15 mL of dried CH_3_CN. A nondissolved part was filtered, and the filtrate was concentrated under reduced pressure until a few milliliters. **K**^**W9Mo2**^_**Sn**_ was precipitated from the latest concentrated solution by adding an excess of absolute ethanol (45 mL). After centrifugation, the product was dried by diethyl ether. After a last centrifugation step, **K**^**W9Mo2**^_**Sn**_ was obtained as a greenish-white powder. Yield: 316 mg (46%). ^1^H NMR (CD_3_CN): δ 7.84 (d + dd, ^3^*J*_H–H_ = 8.13 Hz, ^4^*J*_Sn–H_ = 31.47 Hz, 2H, Ar-*H*), 7.44 (d + dd, ^3^*J*_H–H_ = 8.13 Hz, ^3^*J*_Sn–H_ = 95.4 Hz, 2H, Ar-*H*), 3.13 (m, 32H, N-C*H*_2_-CH_2_-CH_2_-CH_3_), 1.63 (m, 32H, N-CH_2_-C*H*_2_-CH_2_-CH_3_), 1.39 (sex, ^3^*J*_H–H_ = 7,32 Hz, 32H, N-CH_2_-CH_2_-C*H*_2_-CH_3_), 0.98 (t, ^3^*J*_H–H_ = 7.32 Hz, 48H, N-CH_2_-CH_2_-CH_2_-C*H*_3_). ^31^P NMR (CD_3_CN): δ −9.87 (s + d, ^2^*J*_Sn–P_ = 27.07 Hz). IR (KBr, cm^–1^): ν 2962 (m), 2937 (m), 2873 (m), 1481 (m), 1379 (w), 1070 (s), 960 (s), 885 (s), 808 (s), 683(w), 513 (w), 380 (m). Anal. Calcd for PW_9_Mo_2_O_39_SnIC_70_H_148_N_4_: C, 22.16; H, 3.93; N, 1.48. Found: C, 21.95; H, 4.00; N, 1.44. HRMS (ESI^–^, *m*/*z*) for PW_9_Mo_2_O_39_SnIC_70_H_148_N_4_: [M]^4–^, calcd 705.75, found 705,75; [M + TBA]^3–^, calcd 1021.76, found 1022.09; [M + 2TBA]^2–^, calcd 1653.78, found 1654.58.

#### Synthesis of TBA_4_[PW_9_Mo_2_O_39_{Sn(C_6_H_4_)C≡C(C_5_H_4_)Fe(C_5_H_5_)}] (**K**^**W9Mo2**^_**Sn**_**[Fc]**)

**K^W9Mo2^_Sn_** (100 mg, 0.026 mmol), ethynylferrocenyl (16.8 mg, 0.080 mmol), and bis(triphenylphosphine)palladium(II) dichloride (3.1 mg, 0.004 mmol) were dissolved into 2 mL of anhydrous DMF, and the solution was purged with Ar. Freshly distilled NEt_3_ (70 μL) was then added. The mixture was stirred overnight after being degassed with Ar for 5 min more. An unidentified solid was eliminated by centrifugation. A product was precipitated by the addition of diethyl ether (45 mL) to the supernatant. The resulting solid was separated by centrifugation and then solubilized into a minimum of CH_3_CN. TBABr (126 mg, 0.391 mmol) was added to the latest concentrated solution, and the mixture was filtered to eliminate an unidentified solid. **K**^**W9Mo2**^_**Sn**_**[Fc]** was precipitated by the addition of absolute ethanol (45 mL) to the filtrate. The product was separated by centrifugation and dried by the addition of diethyl ether. Pure **K**^**W9Mo2**^_**Sn**_**[Fc]** was obtained after one last centrifugation step. If the number of TBA determined by ^1^H NMR was lower than expected, one more step was required by using TBA-enriched Amberlyst resin in CH_3_CN. The final product was then obtained as an orange powder. Yield: 78.3 mg (77.7%). ^1^H NMR (CD_3_CN): δ 7.66 (d + dd, ^3^*J*_H–H_ = 8.24 Hz, ^3^*J*_Sn–H_ = 95.0 Hz, 2H, Ar-*H*), 7.53 (d + dd, ^3^*J*_H–H_ = 8.24 Hz, ^4^*J*_Sn–H_ = 33.4 Hz, 2H, Ar-*H*), 4.54 (t, ^3^*J*_H–H_ = 1.90 Hz, 2H, Cp-*H*), 4.31 (t, ^3^*J*_H–H_ = 1.90 Hz, 2H, Cp-*H*), 4.27 (s, 5H, Cp-*H*), 3.13 (m, 32H, N-C*H*_2_-CH_2_-CH_2_-CH_3_), 1.63 (m, 32H, N-CH_2_-C*H*_2_-CH_2_-CH_3_), 1.39 (sex, ^3^*J*_H–H_ = 7.36 Hz, 32H, N-CH_2_-CH_2_-C*H*_2_-CH_3_), 0.98 (t, ^3^*J*_H–H_ = 7.36 Hz, 48H, N-CH_2_-CH_2_-CH_2_-C*H*_3_). ^31^P NMR (CD_3_CN): δ −9.85 (s + d, ^2^*J*_Sn–P_ = 26.87 Hz). IR (KBr, cm^–1^): ν 2960 (m), 2933 (m), 2872 (m), 1483 (m), 1379 (w), 1070 (s), 1056 (sh.), 959 (s), 887 (m), 799 (vs), 499 (w), 378 (m), 332 (w). Anal. Calcd for PW_9_Mo_2_O_39_SnFe C_82_H_157_N_4_: C, 25.41; H, 4.08; N, 1.45. Found: C, 25.47; H, 4.12; N, 1.52. HRMS (ESI^–^, *m*/*z*) for PW_9_Mo_2_O_39_SnFe C_82_H_157_N_4_: [M]^4–^, calcd 726.27, found 725.77; [M + TBA]^3–^, calcd 1049.12, found 1049.46; [M + 2TBA]^2–^, calcd 1694.83, found 1696.84.

### Computational Details

DFT calculations were performed with the *Gaussian 16* (revision A03)^79^ quantum chemistry package using three different hybrid exchange-correlation functionals, namely, B3LYP-D3,^80−82^ ωB97X-D,^83^ and HSE06.^84^ The LANL2DZ basis set^85^ and associated pseudopotentials were used to describe the Mo, W, Sn, and I atoms, while the Pople-type 6-31G(d,p) basis set^86−88^ was adopted for the remaining atoms. The solvent effects of CH_3_CN were included in both geometry optimizations and energy calculations by means of the IEF-PCM^89^ implicit solvent model, as implemented in *Gaussian 16*. Geometry optimizations were full and without any symmetry restriction, and all of the minima were characterized by the lack of imaginary frequencies. Absorption spectra were simulated using TD-DFT^90,91^ in combination with the HSE06 functional, solvent effects, and the basis set described above.

## Supporting Information Available

IR, ^1^H and ^31^P NMR, and MS spectra, cyclic voltammograms at various sweep rates, chemical reduction attempts, and DFT calculations, including energies for frontier molecular orbitals, TD-DFT-simulated absorption spectra, and Cartesian coordinates (PDF)
