# Supplementary material for: When Identification of the Reduction Sites in Mixed Molybdenum/Tungsten Keggin-Type Polyoxometalate Hybrids Turns Out Tricky
Source: Inorg Chem. 2022 May 12;61(20):7700–9. doi: 10.1021/acs.inorgchem.2c00866 (PMC9234957; doi:10.1021/acs.inorgchem.2c00866)
Supplement: Supplementary file 1 — ic2c00866_si_001.pdf [file ic2c00866_si_001.pdf]

When identification of the reduction sites in mixed Mo/W Keggin-type polyoxometalate hybrids turns out tricky

Maxime Laurans,[a] Michele Mattera,[a] Raphaël Salles,[a] Ludivine K'Bidi,[a] Pierre Gouzerh,[a] Séverine Renaudineau,[a] Florence Volatron,[a] Geoffroy Guillemot,[a] Sébastien Blanchard,[a] Guillaume Izzet,[a] Albert Solé-Daura,[b,] Josep M. Poblet,[b] Anna Proust[a,]**

[a]Sorbonne Université, CNRS, Institut Parisien de Chimie Moléculaire, IPCM, 4 Place Jussieu, F-75005 Paris, France, anna.proust@sorbonne-universite.fr

[b] Department de Química Física I Inorgànica, Universitat Rovira I Virgili, Marcel-lí Domingo 1, 43007 Tarragona, Spain, albert.sole@alumni.urv.cat

Supporting Information

Contents

Synthesis and characterizations of $(TBA)_4[PW_9Mo_2O_{39}\{Sn(C_6H_4I)\}]$ K^{W9Mo2}_{Sn}
Figure S1. ^{1}H NMR (300 MHz) and ^{31}P (121 MHz, framed inset) spectra of K^{W9Mo2}_{Sn} in CD_3CN.
Figure S2. IR spectrum of K^{W9Mo2}_{Sn} (KBr pellet)
Figure S3. Comparison of experimental (lower trace) and calculated (upper trace) isotopic peaks for the ions $[PW_9Mo_2O_{39}\{Sn(C_6H_4I)\}]^{4-}$ $[PW_9Mo_2O_{39}\{Sn(C_6H_4I)\}.TBA]^{3-}$ and $[PW_9Mo_2O_{39}\{Sn(C_6H_4I)\}.2TBA]^{2-}$ of K^{W9Mo2}_{Sn}

Synthesis and characterizations of $(TBA)_4[PW_9Mo_2O_{39}\{Sn(C_6H_4)C{\equiv}C(C_6H_4)Fc\}]$ K^{W9Mo2}_{Sn}[Fc]
Figure S4. ^{1}H NMR (300 MHz) and ^{31}P (121 MHz, framed inset) spectra of K^{W9Mo2}_{Sn}**[Fc]** in CD_3CN.
Figure S5. IR spectrum of K^{W9Mo2}_{Sn}**[Fc]** (KBr pellet)
Figure S6. Comparison of experimental (lower trace) and calculated (upper trace) isotopic peaks for the ions $[PW_9Mo_2O_{39}\{Sn(C_6H_4)C{\equiv}C(C_6H_4)Fc\}]^{4-}$, $[PW_9Mo_2O_{39}\{Sn(C_6H_4)C{\equiv}C(C_6H_4)Fc\}.TBA]^{3-}$ and $[PW_9Mo_2O_{39}\{Sn(C_6H_4)C{\equiv}C(C_6H_4)Fc\}.2TBA]^{2-}$ of K^{W9Mo2}_{Sn}**[Fc]**

Electrochemical Characterization
Figure S7. Cyclic voltammograms of 0.1 mM K^{W9Mo2}_{Sn} in CH_3CN (0.1 M $TBAPF_6$) at a glassy carbon electrode, potentials given versus SCE electrode, at various scan rates v = 5 (mauve), 10 (mustard yellow), 20 (blue), 50 (balck), 100 (red), 200 (green) et 500 (orange) mV/s.
Figure S8. Cyclic voltammograms of 0.1 mM K^{W9Mo2}_{Sn}**[Fc]** in CH_3CN (0.1 M $TBAPF_6$) at a glassy carbon electrode, potentials given versus SCE electrode, at various scan rates v = 20 (black) 50 (red), 100 (blue), 200 (green) et 400 (orange) mV/s

Chemical reduction of $(TBA)_4[PW_9Mo_2O_{39}\{Sn(C_6H_4I)]$ K^{W9Mo2}_{Sn} and characterization of the 1e reduced $[PW_9Mo_2O_{39}\{Sn(C_6H_4I)]^{5-}$ (I)

Figure S9. Left: UV-Vis-NIR spectra of a 6 10^{-4} M solution of the as obtained **0** + **I** solid mixture redissolved in CH_3CN; right: X-band ESR spectrum of a frozen (20 K) aliquot of the mother solution taken before evaporation to dryness.

DFT calculations

Table S1. Energies for frontier MOs (eV) of K^{W9Mo2}_{Sn} calculated with different density functionals.

Figure S10. Time-dependent DFT-simulated absorption spectra for $[PW_9Mo_2O_{39}\{Sn(C_6H_4I)]^{5-}$ **(1e-K^{W9Mo2}_{Sn}, I**) (left) and $[PW_9Mo_2O_{39}\{Sn(C_6H_4I)]^{6-}$ **(2e-K^{W9Mo2}_{Sn}, II**) (right).

Cartesian coordinates (Å) for the most representative structures optimized at the B3LYP-D3 level.

References

Synthesis and characterizations of $(TBA)_4[PW_9Mo_2O_{39}\{Sn(C_6H_4I)\}]$ $\mathbf{K^{W9Mo2}_{Sn}}$

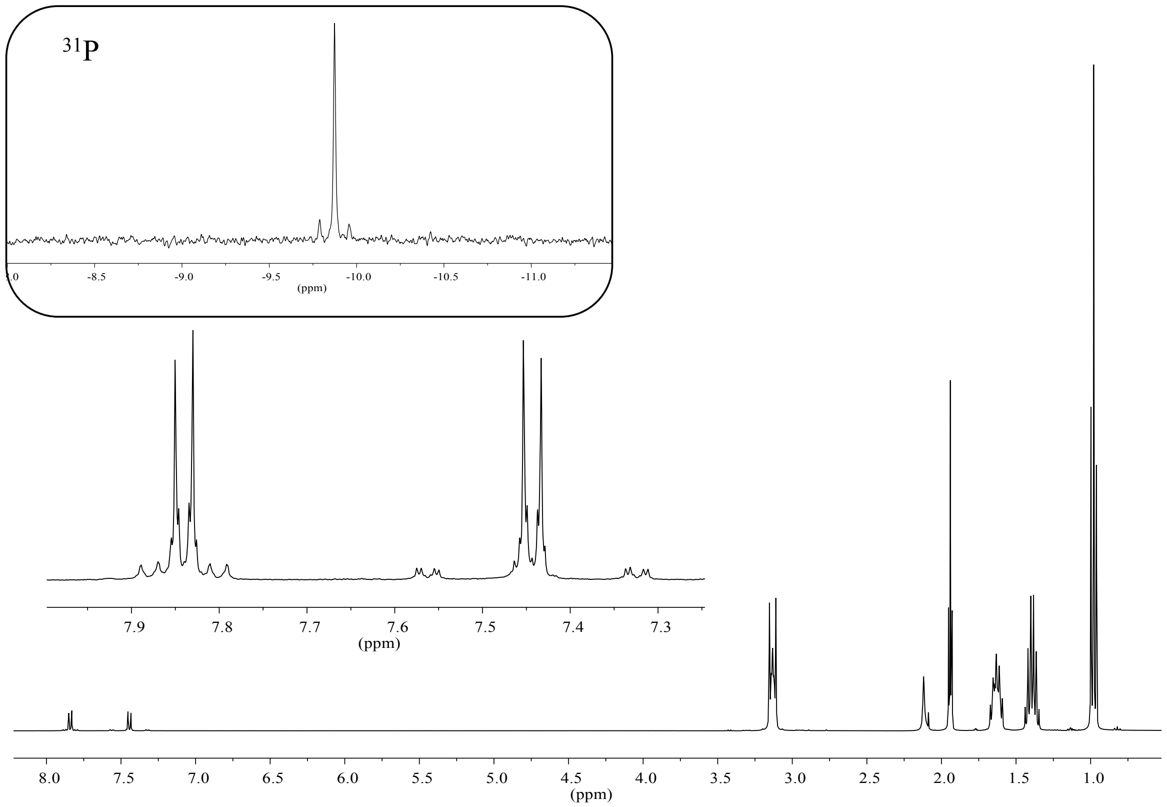

Figure S1. ^{1}H NMR (300 MHz) and ^{31}P (121 MHz, framed inset) spectra of $\mathbf{K^{W9Mo2}_{Sn}}$ in CD_3CN.

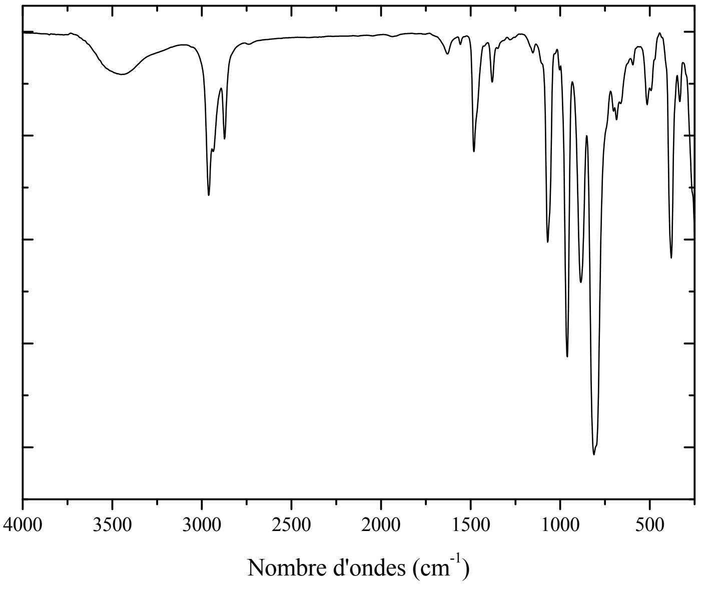

Figure S2. IR spectrum of $\mathbf{K^{W9Mo2}_{Sn}}$ (KBr pellet)

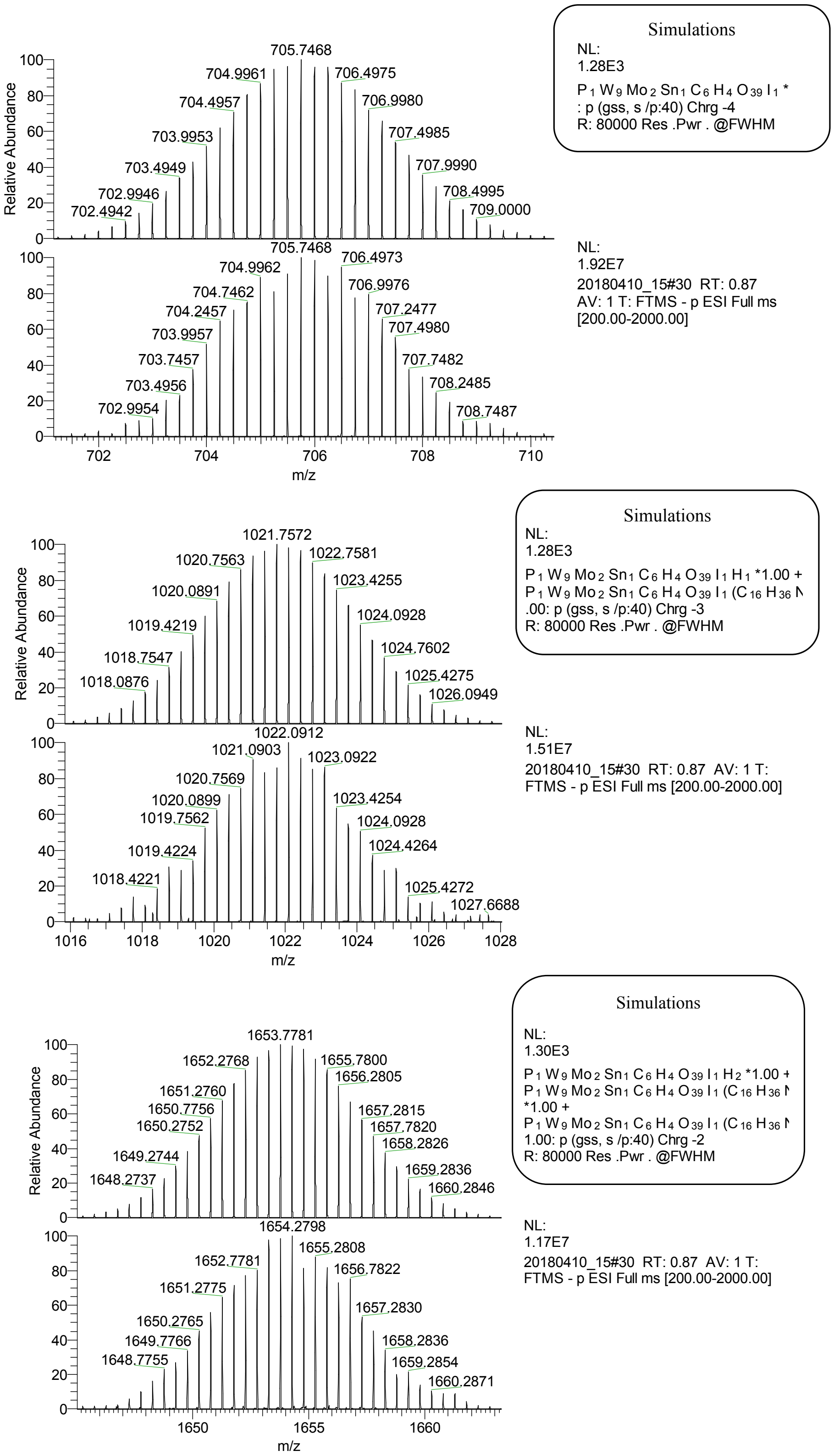

Figure S3. Comparison of experimental (lower trace) and calculated (upper trace) isotopic peaks for the ions $[PW_9Mo_2O_{39}\{Sn(C_6H_4I)\}]^{4-}$ $[PW_9Mo_2O_{39}\{Sn(C_6H_4I)\}.TBA]^{3-}$ and $[PW_9Mo_2O_{39}\{Sn(C_6H_4I)\}.2TBA]^{2-}$ of $\mathbf{K^{W9Mo2}}_{\mathbf{Sn}}$

Synthesis and characterizations of $(TBA)_4[PW_9Mo_2O_{39}\{Sn(C_6H_4)C{\equiv}C(C_6H_4)Fc\}]$ $\mathbf{K^{W9Mo2}_{Sn}[Fc]}$

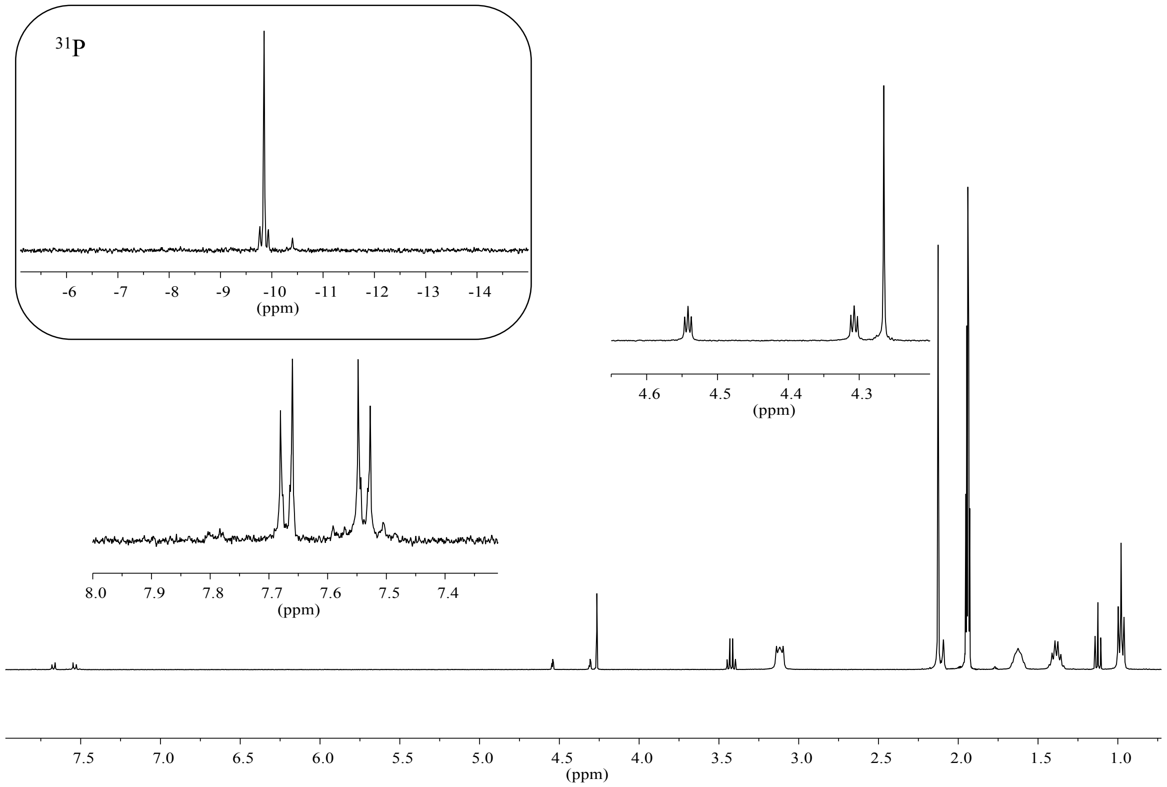

Figure S4. ^{1}H NMR (300 MHz) and ^{31}P (121 MHz, framed inset) spectra of $\mathbf{K^{W9Mo2}_{Sn}[Fc]}$ in CD_3CN.

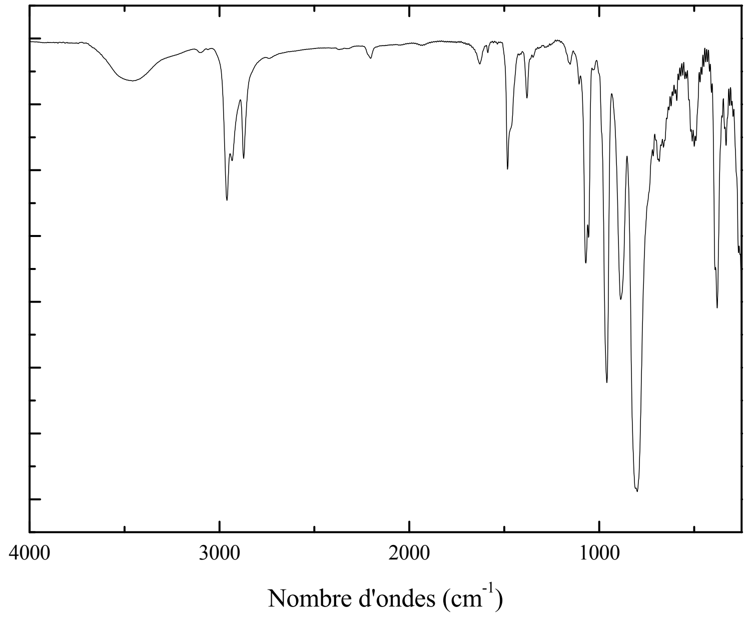

Figure S5. IR spectrum of $\mathbf{K^{W9Mo2}_{Sn}[Fc]}$ (KBr pellet)

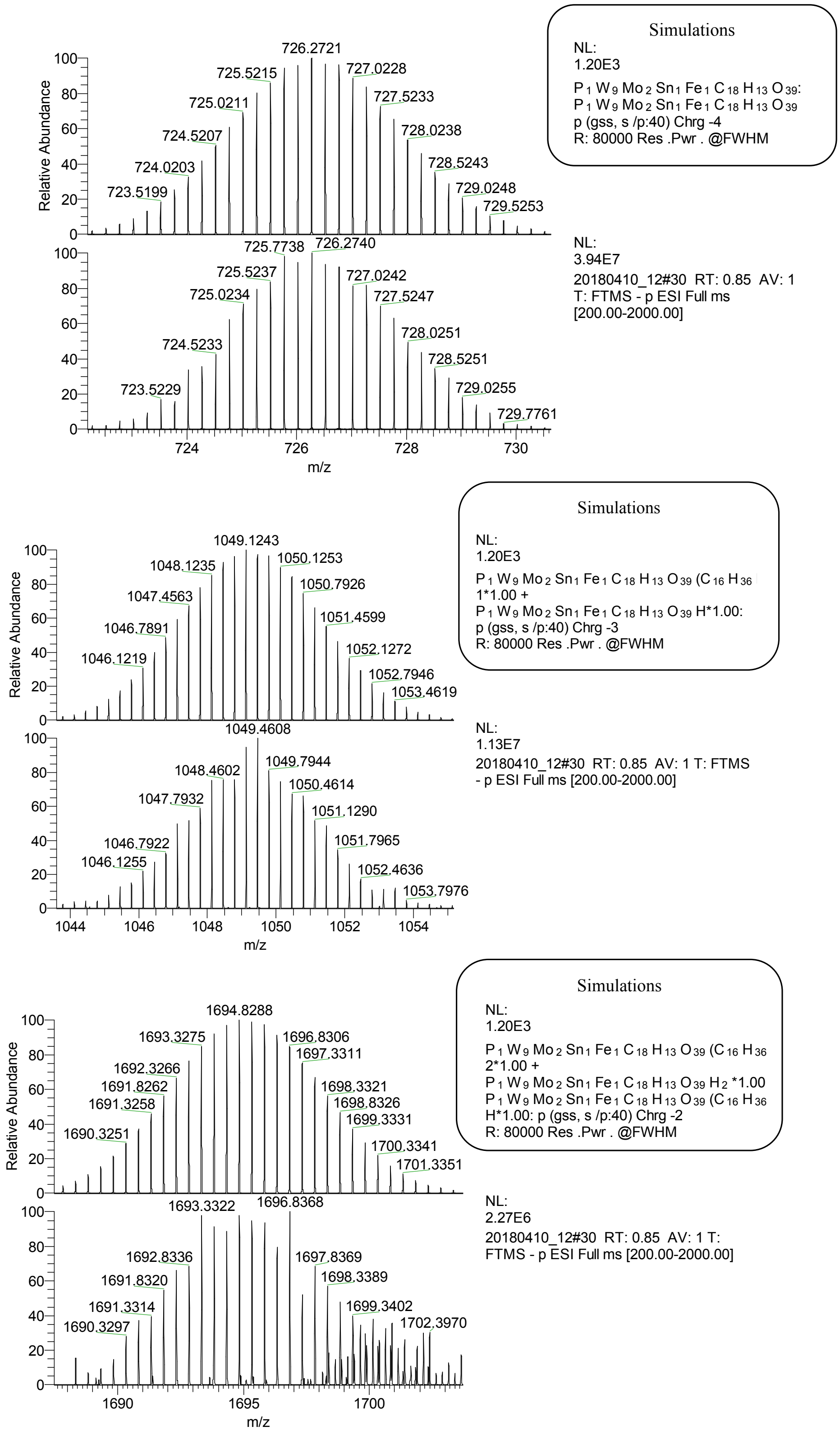

Figure S6. Comparison of experimental (lower trace) and calculated (upper trace) isotopic peaks for the ions $[PW_9Mo_2O_{39}\{Sn(C_6H_4)C{\equiv}C(C_6H_4)Fc\}]^{4-}$, $[PW_9Mo_2O_{39}\{Sn(C_6H_4)C{\equiv}C(C_6H_4)Fc\}.TBA]^{3-}$ and $[PW_9Mo_2O_{39}\{Sn(C_6H_4)C{\equiv}C(C_6H_4)Fc\}.2TBA]^{2-}$ of $\mathbf{K^{W9Mo2}{}_{Sn}[Fc]}$.

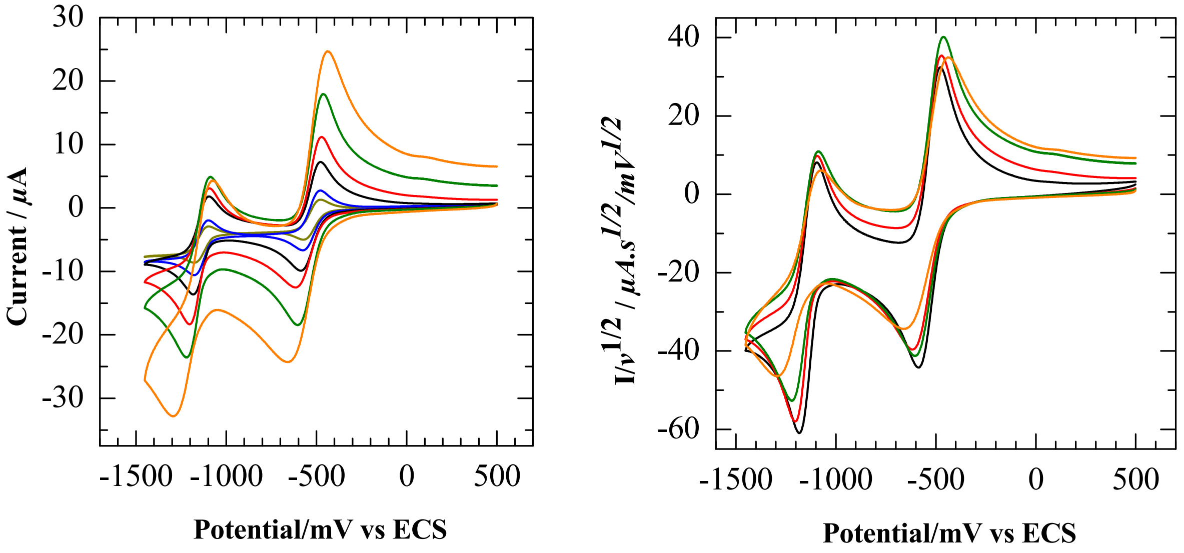

Figure S7. Cyclic voltammograms of 0.1 mM $\mathbf{K^{W9Mo2}_{Sn}}$ in CH_3CN (0.1 M $TBAPF_6$) at a glassy carbon electrode, potentials given versus SCE electrode, at various scan rates v = 5 (mauve), 10 (mustard yellow), 20 (blue), 50 (balck), 100 (red), 200 (green) et 500 (orange) mV/s.

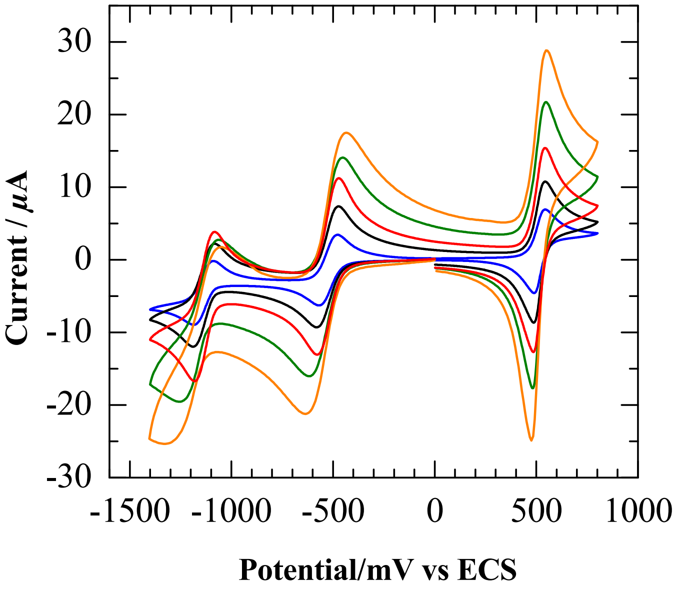

Figure S8. Cyclic voltammograms of 0.1 mM $\mathbf{K^{W9Mo2}_{Sn}}$**[Fc]** in CH_3CN (0.1 M $TBAPF_6$) at a glassy carbon electrode, potentials given versus SCE electrode, at various scan rates v = 20 (black) 50 (red), 100 (blue), 200 (green) et 400 (orange) mV/s

Chemical reduction of $(TBA)_4[PW_9Mo_2O_{39}\{Sn(C_6H_4I)\}]$ K^{W9Mo2}_{Sn} and characterization of the 1e reduced $[PW_9Mo_2O_{39}\{Sn(C_6H_4I)\}]^{5-}$ (I)

A 0.37 M solution of sodium naphtalenide NaNID has been prepared by addition of metallic sodium to naphtalene, as previously described.[1]

First, reduction of $\mathbf{K^{W9Mo2}_{Sn}}$(**0**) in solution in dry and degassed CD_3CN has been monitored directly in a ^{31}P NMR tube equipped with a Young valve by adding successive aliquots of the NaNID solution. The characteristic peak at -9.87 ppm progressively decreased with simultaneous increase of a new peak at -7.67 ppm, that we ascribed to the 1e-reduced $\mathbf{K^{W9Mo2}_{Sn}}$ (**I**). The deshielding of the signal of the 1e-reduced species parallels that observed for $[PMo_{12}O_{40}]^{3-}$.[2] Curiously, no other signal could be detected upon further addition of one equivalent of NaNID.
Then, several trials have been made to prepare the 2e- $\mathbf{K^{W9Mo2}_{Sn}}$ (**II**) in a Schlenk tube by addition of 2 equivalents of the NaNID solution to a solution of $\mathbf{K^{W9Mo2}_{Sn}}$ (**0**) in dry and degassed CH_3CN. However, after evaporation to dryness, the ^{31}P NMR spectrum of the blue solid redissolved in dry and degassed CD_3CN disclosed a mixture of **0** and **I**. At this stage we do not understand the reason of this apparent difficulty in getting **II**. A competitive reduction path involving the iodo-aryl function might be proposed. But the cyclo-votammograms of $\mathbf{K^{W9Mo2}_{Sn}}$ and the UV-Vis spectra obtained by spectro-electrochemistry are characteristic of (multiply) reduced POMs and do not point out the reduction of an organic moiety, that we have never observed in similar compounds. The use of a reducing agent milder than NaNID is probably advisable.

The UV-Vis-NIR and ESR spectra of the mixture of **0** and **I** obtained by chemical reduction are presented below. They display features analogous to that described in the manuscript for solution of **I** in CH_3CN ($TBAPF_6$ 0.1 M), obtained by electrochemical reduction. The observed shift of the wavelength at the maximum absorption can probably be explained by the difference in the environment (presence of sodium cation throughout the chemical reduction and of the supporting electrolyte during the electrochemical reduction), which is known to affect IVCT processes.

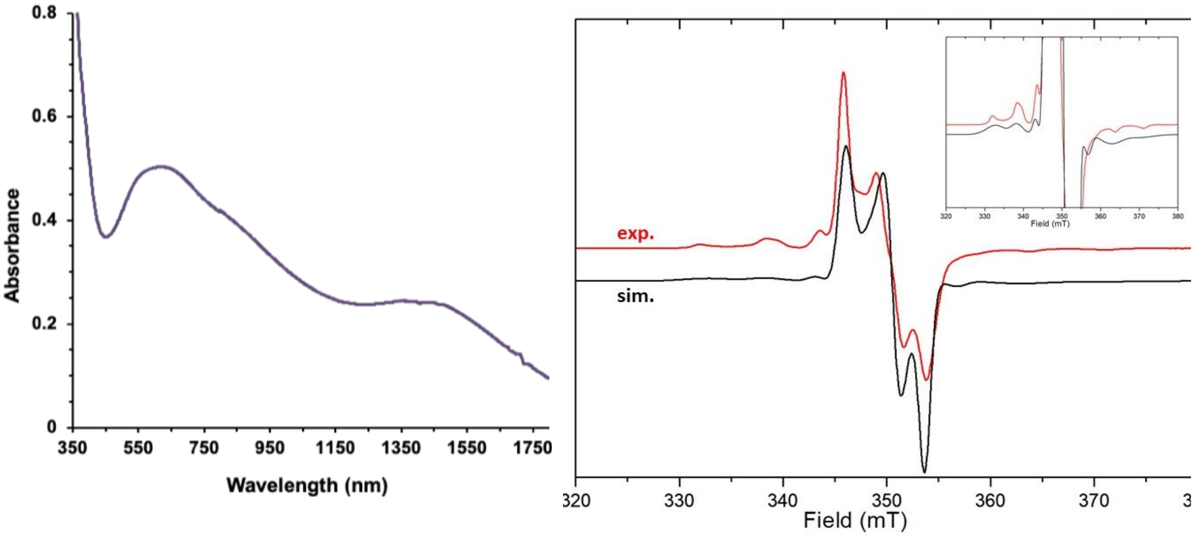

Figure S9. Left: UV-Vis-NIR spectra of a 6 10^{-4} M solution of the as obtained **0** + **I** solid mixture redissolved in CH_3CN; right: X-band ESR spectrum of a frozen (20 K) aliquot of the mother solution taken before evaporation to dryness.

DFT Calculations

Table S1. Energies for frontier MOs (eV) of $K^{W9Mo2}{}_{Sn}$ calculated with different density functionals.

MO	Functional		
	B3LYP-D3	ωB97X-D	HSE06
HOMO	-6.50	-8.52	-6.42
LUMO	-3.37	-1.41	-3.67
LUMO+1	-2.99	-1.06	-3.27
LUMO+2	-2.83	-0.93	-2.99
LUMO+3	-2.77	-0.86	-2.98

DFT simulation of the absorption spectra of $[PW_9Mo_2O_{39}\{Sn(C_6H_4I)]^{5-}$ **(1e-$K^{W9Mo2}{}_{Sn}$, I)** and $[PW_9Mo_2O_{39}\{Sn(C_6H_4I)]^{6-}$ **(2e-$K^{W9Mo2}{}_{Sn}$, II)**

The spectra of species **I** and **II** were simulated by means of time-dependent DFT using the HSE06 functional, which provides the least overestimated HOMO-LUMO gap, along with rather well-reproduced reduction potentials (see Table S1 and Table 2 in the main text). As shown in Figure S17 (left), the shape of the absorption spectrum of **I** matches rather well with the experimental one (Figure 3), although all bands are slightly blue-shifted, presumably due to the overestimated band gap typical of hybrid functionals. The spectrum shows two bands in the visible region (centered at 475 and 603 nm) that can be attributed to the experimental band at 560 nm and the broader shoulder around 720 nm; in addition to another band in the near infrared region, accounting for a SOMO→LUMO excitation (Mo→Mo). When moving to the spectrum of **II (**Figure S17, right), the latter disappears as the low energy-lying d(Mo) orbitals are both occupied. Moreover, in agreement with the evolution of the experimental absorption spectrum upon reduction, the intensity of the bands in the visible region increases due to the contribution of two Mo(V) ions to excitations of Mo→W character. The appearance of an intense band of Mo→W character at 683 nm can be attributed to the band centered at 720 nm in the experimental spectrum, while the less intense band at 491 nm might correspond to the broad shoulder centered at ca. 560 nm (Figure 3).

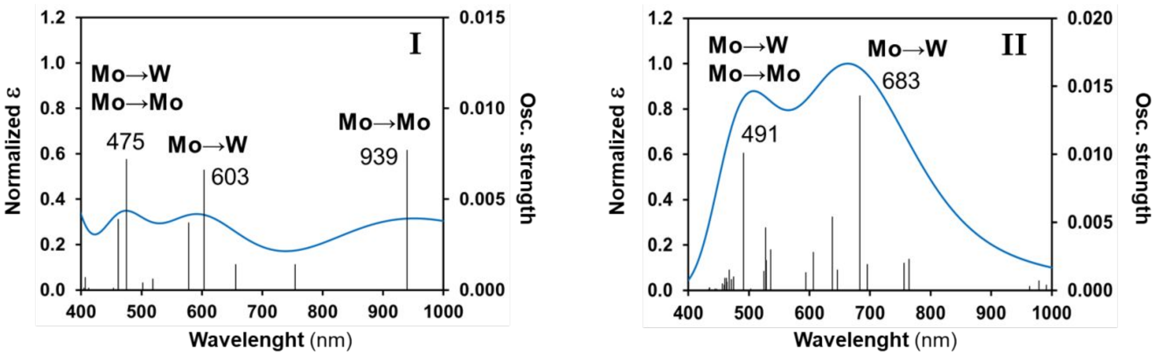

Figure S10. Time-dependent DFT-simulated absorption spectra for $[PW_9Mo_2O_{39}\{Sn(C_6H_4I)]^{5-}$ **(1e-$K^{W9Mo2}{}_{Sn}$, I)** (left) and $[PW_9Mo_2O_{39}\{Sn(C_6H_4I)]^{6-}$ **(2e-$K^{W9Mo2}{}_{Sn}$, II)** (right).

Cartesian coordinates (Å) for the most representative structures optimized at the B3LYP-D3 level.

$[PW_9Mo^{VI}_2O_{39}\{Sn(C_6H_4I)\}]^{4-}$ (K^{W9Mo2}_{Sn})

P	-0.190600	-0.003299	-0.015541
W	-0.187659	-0.297123	3.589936
W	3.425859	-0.170218	-0.105585
W	-0.113923	0.163049	-3.622410
W	1.888213	2.561986	-1.603246
W	1.332600	-2.592641	-2.011218
W	1.425537	-2.742115	1.736483
W	1.918057	2.272060	1.873421
O	-1.441102	0.940014	0.054597
O	-0.227110	-0.954852	1.201509
O	1.118464	0.825881	0.010194
O	-0.260269	-0.807246	-1.333356
O	-2.008703	-2.948836	-0.178458
O	0.134617	2.922412	1.779702
O	1.295468	-3.114610	-0.187300
O	0.079637	3.089203	-1.526118
O	1.287246	0.907945	2.982621
O	-1.355540	1.437185	-2.902955
O	2.726229	-1.426345	1.151378
O	-3.219814	-0.941927	-1.366075
O	2.664500	-1.291989	-1.445058
O	-3.142035	-1.076810	1.368077
O	1.252747	1.261495	-2.949172
O	-1.294480	1.160220	3.032704
O	-1.902254	3.549818	0.174513
O	-0.307694	-3.453614	1.925214
O	2.218431	3.208172	0.141233
O	-0.427211	-3.224247	-2.296005
O	-3.458971	1.921138	-1.289654
O	3.391306	1.155714	1.302578
O	-1.603102	-1.168698	-3.559441
O	1.151235	-1.709569	3.314369
O	-1.498638	-1.574601	3.391750
O	1.019687	-1.379020	-3.524296
O	-3.419054	1.821719	1.547249
O	3.343330	1.312306	-1.334018
O	-0.109410	-0.085385	5.290359
O	5.090006	-0.576056	-0.162284
O	-0.110378	0.520727	-5.298187
O	-2.244388	3.888314	3.002361
O	2.617125	3.757705	-2.592234
O	2.290122	-3.809961	-2.746551
O	-3.131810	-3.722903	2.287899
O	2.422354	-4.020904	2.294181
O	-3.246660	-3.342156	-2.789194
O	-2.355791	4.122863	-2.590739
O	2.684977	3.355296	2.960075
Sn	-3.638422	0.426049	0.091913
C	-5.735505	0.163368	0.126790
C	-6.325182	-0.941220	-0.506345
C	-6.562565	1.077692	0.794719
C	-7.710009	-1.133556	-0.480043
H	-5.706466	-1.665506	-1.030116
C	-7.950004	0.904336	0.830412

H	-6.130751	1.937615	1.300492
C	-8.503930	-0.202981	0.189662
H	-8.151255	-1.992319	-0.973478
H	-8.576442	1.620411	1.350399
I	-10.640880	-0.485300	0.237665
W	-1.868257	2.802907	-1.612436
W	-1.798863	2.659870	1.893441
Mo	-2.150499	-2.231583	-2.099852
Mo	-2.081231	-2.550492	1.621375

$[PW_9Mo^VMo^{VI}O_{39}\{Sn(C_6H_4I)\}]^{5-}$ (I)

P	-0.186816	-0.020345	-0.033659
W	-0.139212	-0.292506	3.581016
W	3.441092	-0.157282	-0.071950
W	-0.214420	0.095585	-3.638632
W	1.846201	2.486404	-1.631421
W	1.371695	-2.571771	-2.057413
W	1.430845	-2.754300	1.699941
W	1.895930	2.315866	1.834144
O	-1.434292	0.927444	0.037608
O	-0.217187	-0.962038	1.193000
O	1.118519	0.823133	-0.017655
O	-0.264457	-0.849674	-1.337029
O	-2.070586	-2.970375	-0.110903
O	0.096295	2.916720	1.782328
O	1.344473	-3.103878	-0.191174
O	0.060221	3.098699	-1.480209
O	1.277944	0.919297	2.958837
O	-1.358872	1.477857	-2.916677
O	2.744304	-1.409451	1.186180
O	-3.229301	-0.919130	-1.426503
O	2.703262	-1.263362	-1.413880
O	-3.156183	-1.069539	1.417567
O	1.215502	1.229582	-2.898001
O	-1.308046	1.133259	3.030552
O	-1.937969	3.538582	0.193909
O	-0.297900	-3.457728	1.930147
O	2.193112	3.204033	0.141378
O	-0.313363	-3.271994	-2.324480
O	-3.468358	1.930188	-1.290720
O	3.380465	1.177609	1.331343
O	-1.579991	-1.157021	-3.599998
O	1.164664	-1.713085	3.304245
O	-1.474863	-1.568954	3.410606
O	1.094645	-1.339385	-3.530899
O	-3.438225	1.759933	1.514004
O	3.367297	1.326557	-1.300289
O	-0.050210	-0.068108	5.284545
O	5.114921	-0.551452	-0.108436
O	-0.138187	0.511195	-5.305752
O	-2.312026	3.825337	3.011582
O	2.572516	3.692528	-2.621814
O	2.394693	-3.756630	-2.771468
O	-3.126253	-3.713455	2.383295
O	2.431611	-4.031629	2.269988
O	-3.182522	-3.478353	-2.754585

O	-2.357899	4.145609	-2.573320
O	2.642548	3.405277	2.936844
Sn	-3.620957	0.354941	0.026978
C	-5.735934	0.158644	0.102331
C	-6.371927	-0.880973	-0.593762
C	-6.529000	1.045160	0.845485
C	-7.761660	-1.037340	-0.557882
H	-5.780515	-1.582769	-1.176989
C	-7.920766	0.910220	0.894323
H	-6.062110	1.855437	1.399775
C	-8.516777	-0.133546	0.188223
H	-8.236044	-1.846584	-1.102085
H	-8.518415	1.606047	1.472791
I	-10.663430	-0.356298	0.252487
W	-1.873785	2.806312	-1.609616
W	-1.839996	2.612397	1.887917
Mo	-2.135605	-2.348484	-2.021780
Mo	-2.087805	-2.543023	1.683522

$[PW_9Mo^V{}_2O_{39}\{Sn(C_6H_4I)\}]^{6-}$ (II)

P	-0.177058	-0.061050	-0.006869
W	-0.086966	-0.321318	3.600747
W	3.438714	-0.130633	-0.087060
W	-0.238262	0.056320	-3.623098
W	1.810224	2.514490	-1.659933
W	1.394701	-2.576341	-2.059747
W	1.475875	-2.772927	1.703256
W	1.882000	2.332816	1.821282
O	-1.435406	0.875277	0.067086
O	-0.188688	-1.007059	1.220985
O	1.116481	0.802260	0.011828
O	-0.243304	-0.873858	-1.325456
O	-1.927579	-3.006348	-0.121751
O	0.074849	2.911937	1.779326
O	1.438258	-3.104711	-0.201422
O	0.009515	3.083634	-1.481276
O	1.303654	0.941080	2.958428
O	-1.399054	1.426036	-2.890915
O	2.783044	-1.364952	1.170609
O	-3.273799	-0.970188	-1.434166
O	2.724874	-1.222804	-1.439890
O	-3.202487	-1.119279	1.461043
O	1.186198	1.242318	-2.905912
O	-1.279839	1.111468	3.059070
O	-1.980242	3.485391	0.217879
O	-0.164121	-3.539215	1.955499
O	2.143192	3.232382	0.117660
O	-0.255953	-3.313769	-2.325207
O	-3.498364	1.840996	-1.235036
O	3.387887	1.236937	1.306017
O	-1.571040	-1.203277	-3.584609
O	1.252962	-1.696117	3.310134
O	-1.418628	-1.577465	3.489113
O	1.107255	-1.339497	-3.535948
O	-3.448554	1.694693	1.547576
O	3.336117	1.377689	-1.321579

O	0.043289	-0.068634	5.302781
O	5.127110	-0.487005	-0.141092
O	-0.177723	0.485646	-5.293459
O	-2.335983	3.770578	3.035857
O	2.504838	3.738326	-2.659819
O	2.452247	-3.729799	-2.788899
O	-3.053252	-3.833897	2.440479
O	2.559504	-3.994867	2.262872
O	-3.163061	-3.560476	-2.706059
O	-2.446625	4.065016	-2.538950
O	2.620262	3.448451	2.912242
Sn	-3.630390	0.233435	0.085500
C	-5.768220	0.161040	0.099409
C	-6.444248	-0.819037	-0.644965
C	-6.538744	1.066887	0.844232
C	-7.841285	-0.901383	-0.654282
H	-5.876211	-1.535528	-1.234324
C	-7.937223	1.008196	0.851829
H	-6.045101	1.835452	1.434161
C	-8.567827	0.019471	0.098445
H	-8.342300	-1.666685	-1.237179
H	-8.513688	1.718870	1.434113
I	-10.727145	-0.090535	0.099483
W	-1.925082	2.734280	-1.573463
W	-1.855678	2.551890	1.913741
Mo	-2.142158	-2.402790	-1.955152
Mo	-2.060868	-2.602429	1.772742

References

(1) Zhang, T.; Solé-Daura, A.; Hostachy, S.; Blanchard, S.; Paris, C.; Li, Y.; Carbó, J. J.; Poblet, J. M.; Proust, A.; Guillemot, G. Modeling the Oxygen Vacancy at a Molecular Vanadium(III) Silica-Supported Catalyst. *J. Am. Chem. Soc.* **2018**, *140* (44), 14903–14914. https://doi.org/10.1021/jacs.8b09048.

(2) Artero, V.; Proust, A. Reduction of the Phosphododecamolybdate Ion by Phosphonium Ylides and Phosphanes. *Eur. J. Inorg. Chem.* **2000**, *2000* (11), 2393–2400. https://doi.org/10.1002/1099-0682(200011)2000:11<2393::AID-EJIC2393>3.0.CO;2-G.
